# Assessing secondhand and thirdhand tobacco smoke exposure in Canadian infants using questionnaires, biomarkers, and machine learning

**DOI:** 10.1038/s41370-021-00350-4

**Published:** 2021-06-26

**Authors:** Jaclyn Parks, Kathleen E. McLean, Lawrence McCandless, Russell J. de Souza, Jeffrey R. Brook, James Scott, Stuart E. Turvey, Piush J. Mandhane, Allan B. Becker, Meghan B. Azad, Theo J. Moraes, Diana L. Lefebvre, Malcolm R. Sears, Padmaja Subbarao, Tim K. Takaro

**Affiliations:** 1 Faculty of Health Sciences, Simon Fraser University, Burnaby, BC Canada; 2 BC Centre for Disease Control, Vancouver, BC Canada; 3 Department of Health Research Methods, Evidence, and Impact, Faculty of Health Sciences, McMaster University, Hamilton, ON Canada; 4 Dalla Lana School of Public Health, University of Toronto, Toronto, ON Canada; 5 Department of Pediatrics, Faculty of Medicine, University of British Columbia, Vancouver, BC Canada; 6 Department of Pediatrics, University of Alberta, Edmonton, AB Canada; 7 Department of Pediatrics and Child Health, University of Manitoba, Winnipeg, MB Canada; 8 Children’s Hospital Research Institute of Manitoba, Winnipeg, MB Canada; 9 Hospital for Sick Children, Toronto, ON Canada; 10 Department of Pediatrics, University of Toronto, Toronto, ON Canada; 11 Department of Medicine, Faculty of Health Sciences, McMaster University, Hamilton, ON Canada

**Keywords:** Childhood asthma, Biomarker, Secondhand smoke, Cotinine, Thirdhand smoke, Variable importance

## Abstract

**Background:**

As smoking prevalence has decreased in Canada, particularly during pregnancy and around children, and technological improvements have lowered detection limits, the use of traditional tobacco smoke biomarkers in infant populations requires re-evaluation.

**Objective:**

We evaluated concentrations of urinary nicotine biomarkers, cotinine and *trans*-3’-hydroxycotinine (3HC), and questionnaire responses. We used machine learning and prediction modeling to understand sources of tobacco smoke exposure for infants from the CHILD Cohort Study.

**Methods:**

Multivariable linear regression models, chosen through a combination of conceptual and data-driven strategies including random forest regression, assessed the ability of questionnaires to predict variation in urinary cotinine and 3HC concentrations of 2017 3-month-old infants.

**Results:**

Although only 2% of mothers reported smoking prior to and throughout their pregnancy, cotinine and 3HC were detected in 76 and 89% of the infants’ urine (*n* = 2017). Questionnaire-based models explained 31 and 41% of the variance in cotinine and 3HC levels, respectively. Observed concentrations suggest 0.25 and 0.50 ng/mL as cut-points in cotinine and 3HC to characterize SHS exposure. This cut-point suggests that 23.5% of infants had moderate or regular smoke exposure.

**Significance:**

Though most people make efforts to reduce exposure to their infants, parents do not appear to consider the pervasiveness and persistence of secondhand and thirdhand smoke. More than half of the variation in urinary cotinine and 3HC in infants could not be predicted with modeling. The pervasiveness of thirdhand smoke, the potential for dermal and oral routes of nicotine exposure, along with changes in public perceptions of smoking exposure and risk warrant further exploration.

## Introduction

Tobacco smoke exposure has been studied extensively for its negative health effects and is known to be particularly harmful to children [1]. The health effects of firsthand smoking are now well appreciated and research priorities have shifted to understanding secondhand (SHS) and thirdhand smoking (THS) [2–4]. SHS both in utero and in childhood has been repeatedly linked to asthma, as well as sudden infant death syndrome, low birth weight, cancer, dental caries, hearing loss, metabolic syndromes, and a breadth of poor behavioral and cognitive outcomes [3, 5]. SHS occurs as a result of passive exposure to the smoke from the burning end of a cigarette and the smoke breathed out by smokers. THS results from SHS that has been absorbed onto surfaces such as carpeting and upholstery, or settled on dust where it can persist a long period of time and be re-emitted into the air long after a smoking event [6].

Accurately assessing prenatal and early life tobacco smoke exposure is important to understand and reduce childhood asthma and wheeze [7]. Questionnaires are a flexible and relatively inexpensive method of assessing exposure, but biomarkers of tobacco smoke exposure are more accurate, objective, and can be obtained with little burden to the participant using passive urine sample collection. Nicotine is an important component of cigarettes for researchers because it can be reliably detected in humans at concentrations proportional to cigarette smoke exposure [8]. While many metabolites of nicotine can be analyzed, cotinine is the most widely used biomarker of recent tobacco smoke exposure, has an average half-life of 16–19 h in children ages 2 months to 4 years [9, 10], and is heralded as the best biomarker of tobacco smoke exposure [11]. More than 70% of nicotine is metabolically converted into cotinine, which is then metabolically converted into *trans*-3’-hydroxycotinine (3HC) [10].

When smoking prevalence is high, the use of cotinine to measure tobacco smoke exposure aids in verification of the confirmed exposure. However, over the past two decades the rates of smoking in Canada have been decreasing, particularly during pregnancy and in the presence of children. This is a combined result of policy, increased awareness, product labeling, clinician counseling, and social pressures [11–18]. Smoking rates during pregnancy are trending downwards in Canada—from an estimated at 24% in the 1990s [19], to approximately 17% in 2000 [17], and 11% in 2006 [12]. The Canadian Tobacco Use Monitoring Survey reported that in 2012, 22.8% of Canadian women age 20–24 years and 4.8% of women age 25–44 years reported smoking during their most-recent pregnancy [20]. Excluding the Yukon, Northwest territories, and Nunavut, the overall smoking rate among all Canadians over the age of 15 has dropped as well, from 25% in 1999 to 16% in 2012 [21]. Tests with lower levels of detection for cotinine have been put into practice. However, there is limited knowledge of how these refinements may impact the use of this biomarker for public health in low-smoking settings.

The purpose of this study was to better understand cotinine and 3HC as biomarkers of tobacco smoke exposure in a population with little to no reported tobacco smoke exposure. The research questions were “What is the amount of tobacco smoke exposure in a population of urban Canadian infants?”, “What are the key sources or predictors of tobacco smoke exposure?”, and “What are the research implications and the relevance of nicotine biomarker concentrations to policy makers, researchers and clinicians?”. This allows for a better understanding of how biomarker data could be organized within cohorts, and how low-level nicotine metabolites should be considered and interpreted as biomarkers by environmental health researchers and how policy might reduce exposure to infants.

## Materials, participants, and methods

### Participants

This study uses secondary data from the CHILD Cohort Study, a four-center (Vancouver, Edmonton, Manitoba, and Toronto) longitudinal, population-based birth-cohort study that enrolled 3455 mother–child pairs between 2008 and 2012. The main focus of CHILD was to identify environmental and genetic determinants of allergic disorders and asthma. The demographic characteristics of the cohort reflect lower smoking rates relative to the general Canadian population, with a very small proportion of mothers in the study reporting that they smoked during pregnancy or during the child’s early life [22]. Data were collected using a combination of questionnaires, in-home visits, and urine samples. Questionnaires and biological samples pertaining to the growing infants’ exposures, lifestyle, and health were collected at various time points, with many questions repeated to allow for longitudinal analyses [23].

### Sampling design

Ethics approval was obtained through Simon Fraser University, deeming this project to be a minimal risk study [2018s0608]. Research ethics approval for the overall CHILD study was obtained at each recruitment site and through the Hamilton Integrated Ethics Board (certificate 07-2929).

Urine samples were collected by trained research assistants during the 3-month in-home visits. The procedure involved placing a plastic Tegaderm™ film over the wetting area of the baby’s diaper to prevent urine absorption by the diaper. Cotton pads were placed on top of the film and the baby then wore the diaper for the duration of the home visit. At the end of the visit, the mother removed the diaper, and the research assistant placed the cotton pads into a syringe, aliquoted the sample into six vials, and measured the specific gravity (SG) of the sample using a calibrated refractometer. The samples were stored at –80 degrees Celsius [23]. In the laboratory, β-glucuronidase was used to de-conjugate any glucoronidated cotinine and 3HC molecules. After extraction, the samples were analyzed by liquid chromatography-atmospheric pressure chemical ionization tandem mass spectrometry.

Analyte concentrations were calculated using least-squares linear regression of the peak area ratios of native to internal standards. The limit of detection for both cotinine and 3HC was 0.030 ng/mL [24]. To account for dilution of the urine samples, biomarker concentrations were corrected for SG [22, 23]. The following formula was applied to adjust concentrations to a standardized SG: *P*_*C*_ = *P* × [(*SG*_MED_ – 1)/(*SG* – 1)], where *P* is the uncorrected metabolite concentration, *P*_*C*_ is the corrected concentration, *SG*_MED_ is the median urine specific gravity of the study population (1.004) and *SG* is the specific gravity measure of the sample. Samples with SG measurements outside the normal human range (3 standard deviations above the median) were excluded from the analysis. Since the metabolite concentrations were approximately log-normally distributed, the concentrations were log-transformed (base 2) prior to correction for SG (i.e., dilution). Concentrations below the level of detection (LOD) were imputed with a concentration between 0 and the LOD in a way that matched the log-normal distribution of the truncated concentration [25]. Concentrations were determined for each of the two metabolites in ng/mL.

Tobacco smoke exposure can be firsthand, secondhand, thirdhand, or any combination of these. While it cannot be determined based on a measure of biomarker concentration alone whether the participant was exposure to recent THS, or SHS some time ago, questionnaires offer a valuable means of characterizing exposure. The exposure variables were taken from three sources: a questionnaire completed by the mother during pregnancy, a parent-completed household exposures questionnaire at 3 months, and a research assistant-completed questionnaire on household exposures completed at the 3-month home visit. Many questions originally asked about the mother’s environment during pregnancy were asked again during the child’s 3-month questionnaire. Potential predictors of exposure were derived from questionnaires that captured smoking-related exposure, housing characteristics, and demographics that have been linked in the literature to tobacco smoke exposure, be it secondhand, or thirdhand [6, 8, 26–30].

### Statistical methods

Geometric means and 95% confidence intervals of both cotinine and 3HC concentrations were calculated and reported by predictor variable. For normally distributed variables, *t*-tests or one-way analysis of variance (ANOVA) tests were used to assess whether the difference in means between levels or groups of a variable were statistically significant. When an ANOVA test was significant, Tukey Honest Significant Differences tests were run to assess multiple pairwise comparisons between multiple levels of a predictor. ANOVA and *t*-tests assume normality in the distribution of the means being compared. For non-normally distributed variables (determined using a Shapiro–Wilk test), a Wilcoxon test was used in place of a *t*-test, and a Kruskal–Wallis test in place of an ANOVA analysis. Spearman correlation tests were used when comparing the biomarker concentrations to a continuous predictor variable.

A random forest regression (RFR) of all potential predictor variables (specified a priori) against each metabolite concentration produced variable importance scores for each predictor. RFR is a machine learning technique that allows a system to automatically learn from the data and produce results with minimal subjectivity, offering an objective perspective on the variable selection process. The RFR was set to run models on 1000 trees, a relatively large number for RFR, to help ensure that the average model fit best reflected the data and avoids overfitting. These variable importance scores reflected the overall influence of the predictor in a prediction model, by showing how much the mean squared error (MSE) of a model would increase as a result of the predictor being excluded from the model. The MSE is a measure of closeness of a fitted line to actual data points, with values between zero and infinity. Having a well-fit model with variables of high importance will result in a lower MSE.

Using variable importance scores derived from RFR, we created multivariable linear regression (MLR) models to predict urinary concentrations as the outcome. A MLR model was then selected based on questionnaire variables identified as “important,“ and an a priori assessment to best explained the cotinine concentrations of the sample with detectable metabolite data. The models were built using manual selection, starting with the predictor with the highest variable importance score. The predictor with the next highest score was then added to the model. The added predictor was included if it had appropriate directionality, increased the model’s coefficient of determination (*R*^2^), and had a *p* < 0.15, as opposed to the more-restrictive *p* < 0.05. This was repeated for many predictor variables (see Appendix) until it was clear that adding more predictors did not further improve model performance. The coefficient of determination and measures of model fit were then used to determine how well questionnaire-based models explain variation in urinary cotinine concentrations in infants. This process was repeated for 3HC, as some variables may be more important for one metabolite than another. Plots of predicted versus observed biomarker concentrations were created for each final model to assess model fit. Regression coefficients and their 95% confidence intervals were reported for each predictor in the final MLR models against the log-transformed cotinine or 3HC concentration. These coefficients were then inverse-log-transformed to reflect the multiplicative change in urinary concentration (i.e., 1.10 means a 10% increase in concentration). The coefficients and back-transformations from unadjusted regression against the log-transformed urinary concentrations were also calculated for each predictor.

A ten-fold cross validation (CV) was also applied to the final 3HC and cotinine prediction models to evaluate prediction error for both models:

1. The dataset was randomly divided into ten sub-groups, or “folds,” with approximately the same number of observations in each group.
2. The predictive model was parameterized based on data from nine of the ten groups.
3. The estimated coefficients were used to predict log-transformed urinary cotinine (or 3HC) concentrations for observations in the excluded group.
4. Steps 1–3 were repeated to obtain predictions for all ten groups and, therefore, all observations.
5. Log-transformed urinary concentration predictions and measurements on the untransformed scale were compared and model performance was evaluated based on *R*^2^.

Biomarkers can also be used to predict or verify the exposure level of participants. Density plots of the urinary metabolite concentrations by important questionnaire questions were used to examine the separation of the participants by questionnaire response [31]. We then compared density proportions of these responses within cut-point bounds to gain consensus about what average concentration is found in those who likely have no household exposure, those who have some moderate exposure, and those who have concentrations consistent with confirmed household SHS exposure. In some cases, these levels were continuous (e.g., week of gestation that mother quit smoking), while others were factors (e.g., location). Recommended cut-points for urinary cotinine concentrations from previous studies were assessed [32].

Analysis was undertaken using R version 3.5.1 (2018-07-02).

## Results

Of 2017 infants with complete data (Supplementary Fig. 1), 76% had detectable cotinine and 89% had detectable 3HC concentrations. Participants from Manitoba (Winnipeg, Morden, and Winkler) made up the largest proportion of our sample (31%), followed by Vancouver (27%), Edmonton (22%), and Toronto (20%). More than half of our participants had a household income over $100,000/year (55%), lived in a single-family home (56%), and had a mother over age 30 (60%). A minority of the sample (34%) lived in a rented home, and 31% had at least one parent with a history of asthma. Less than 3% reported actively smoking during their pregnancy, while 21% reported being recently exposed to smoke during pregnancy. Nearly 18% of mothers were smokers but had quit prior to their pregnancy. Of the 6% of our sample who did not quit prior to their pregnancy, 64% reported quitting during the pregnancy, leaving just 2% who continued to smoke throughout their pregnancy. Most (62%) of mothers were exclusively breastfeeding their child, 26% were partially breastfeeding, and 12% reported not breastfeeding their child at 3–4 months of age. Only 12% reported that smoking had occurred at the home since the child’s birth, with the majority of household smoking occurring outdoors. Table 1 contains more information of the demographic makeup and self-reported smoke exposure of our sample along with how the urinary concentrations differ based on these characterizations. Metabolite concentrations for additional characteristics can be found in the Supplementary information (Supplementary Table 1a–c), along with their corresponding importance scores (Supplementary Fig. 2a, b and Supplementary Table 2).

**Table 1:** Urinary cotinine and *trans*-3’-hydroxycotinine concentrations by self-reported demographic characteristics and tobacco smoke exposure.

Characteristic	% (*N*)	Geometric mean urinary cotinine (95% CI), ng/mL	Geometric mean urinary *trans*-3’-hydroxycotinine (95% CI), ng/mL
*Study center*			
Vancouver	26.7 (539)	0.10 (0.09–0.12)	0.16 (0.14–0.18)
Edmonton	19.9 (402)	0.13 (0.11–0.15)	0.28 (0.23–0.34)
Winnipeg, Morden, Winkler	30.9 (624)	0.14 (0.12–0.16)	0.29 (0.25–0.34)
Toronto	22.4 (452)	0.09 (0.08–0.10)	0.20 (0.17–0.22)
Difference in means, *p* value		***p*** < 0.001	***p*** < 0.001
*Household Income*			
$0–49,999/year	10.0 (201)	0.26 (0.20–0.34)	0.55 (0.43–0.76)
$50,000–99,999/year	31.3 (631)	0.14 (0.12–0.16)	0.26 (0.22–0.29)
$100,000–149,999/year	26.4 (533)	0.09 (0.08–0.11)	0.19 (0.16–0.21)
$150,000+/year	23.3 (469)	0.08 (0.07–0.09)	0.14 (0.12–0.16)
Prefers to not say	9.1 (183)	0.12 (0.10–0.15)	0.28 (0.22–0.36)
Difference in means, *p* value		***p*** < 0.001	***p*** < 0.001
*Maternal age at enrollment, years*			
17–23	3.8 (77)	0.42 (0.28–0.64)	1.01 (0.64–1.60)
24–30	32.2 (649)	0.14 (0.12–0.16)	0.29 (0.25–0.33)
31–35	41.9 (845)	0.10 (0.09–0.11)	0.19 (0.17–0.21)
36–46	22.1 (446)	0.09 (0.08–0.11)	0.17 (0.15–0.20)
Difference in means, *p* value		***p*** < 0.001	***p*** < 0.001
*Child’s sex*			
Male	52.9 (1067)	0.11 (0.10–0.13)	0.20 (0.18–0.23)
Female	47.1 (950)	0.12 (0.10–0.13)	0.23 (0.20–0.26)
Difference in means, *p* value		*p* = 0.86	*p* = 0.26
*Parental asthma*			
Yes	31.3 (660)	0.13 (0.12–0.15)	0.24 (0.21–0.27)
No	67.3 (1357)	0.11 (0.10–0.12)	0.22 (0.20–0.24)
Difference in means, *p* value		***p*** **=** 0.03	*p* = 0.11
*Rent vs. own home*			
Rent	23.2 (467)	0.19 (0.16–0.22)	0.36 (0.31–0.43)
Own	76.8 (1550)	0.10 (0.09–0.11)	0.19 (0.18–0.21)
Difference in means, *p* value		***p*** < 0.001	***p*** < 0.001
*Dwelling type*			
Single family	55.8 (1470)	0.10 (0.10–0.11)	0.20 (0.18–0.22)
Multi-family or apartment	25.8 (521)	0.15 (0.13–0.18)	0.29 (0.25–0.34)
Trailer or other	1.2 (26)	0.24 (0.11–0.49)	0.53 (0.23–1.19)
Difference in means, *p* value		***p*** < 0.001	***p*** < 0.001
*Breastfeeding status at 3 months*			
None	12.0 (243)	0.16 (0.13–0.19)	0.29 (0.23–0.36)
Partial	25.8 (520)	0.12 (0.10–0.14)	0.24 (0.20–0.28)
Exclusive	62.2 (1254)	0.11 (0.10–0.12)	0.21 (0.19–0.23)
Difference in means, *p* value		***p*** = 0.005	*p* = 0.16
*Someone has smoked at the home since birth*			
No smoking at the home	87.8 (1771)	0.10 (0.09–0.10)	0.17 (0.16–0.19)
Yes, smoking at the home	12.2 (246)	0.50 (0.38–0.65)	1.36 (1.02–1.81)
Difference in means, *p* value		***p*** < 0.001	***p*** < 0.001
*Location of household smoking during child’s early life*			
Inside	0.5 (10)	2.07 (0.79–5.44)	6.45 (2.50–16.63)
Near a window or in garage	1.6 (32)	1.30 (0.63–2.71)	4.18 (1.98–8.85)
Outside	11.2 (225)	0.43 (0.33–0.57)	1.16 (0.86–1.57)
Difference in means, *p* value		***p*** **<** 0.001	***p*** < 0.001
*Mother reports smoke exposure during pregnancy*			
Recent exposure	21.1 (425)	0.29 (0.24–0.35)	0.66 (0.54–0.81)
No recent exposures	78.9 (1592)	0.09 (0.08–0.10)	0.29 (0.24–0.35)
Difference in means, *p* value		***p*** < 0.001	***p*** < 0.001
*Maternal smoking status in pregnancy*			
Never smoked	97.5 (1967)	0.10 (0.10–0.11)	0.20 (0.19–0.21)
Daily or occasional smoker	2.6 (50)	7.13 (4.18–12.14)	21.96 (12.21–39.48)
Difference in means, *p* value		***p*** < 0.001	***p*** < 0.001
*Someone has smoked at the home during pregnancy*			
No smoking at the home	88.9 (1794)	0.10 (0.09–0.10)	0.18 (0.17–0.19)
Yes, smoking at the home	11.1 (223)	0.56 (0.44–0.71)	1.44 (1.11–1.87)
Difference in means, *p* value		***p*** < 0.001	***p*** < 0.001
*Location of household smoking during child’s early life*			
Inside	1.0 (20)	3.00 (1.46–6.15)	8.58 (3.68–19.99)
Near a window or in garage	1.9 (38)	0.91 (0.54–1.55)	2.62 (1.39–4.94)
Outside	8.8 (178)	0.47 (0.36–0.61)	1.19 (0.89–1.59)
Difference in means, *p* value		***p*** **<** 0.001	***p*** < 0.001

The proportion and crude number of sample participants that corresponds to each level of household characteristic variables is reported to the nearest whole number. The geometric mean (95% confidence interval) of the corrected and log-transformed cotinine distribution for each level of each variable is also shown. *P* values indicate whether the difference in log-transformed means was statistically significant (*p* < 0.05) amongst the variable levels based on ANOVA or *t*-tests.

### Urinary concentrations

**Fig. 1:**
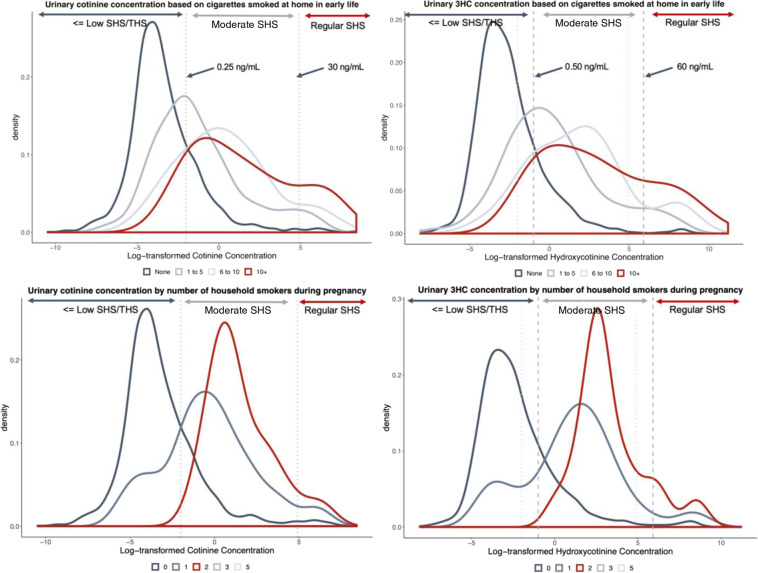
Questionnaire-determined density plots of cotinine and *trans*-3’-hydroxycotinine concentrations. The distribution of the log-transformed urinary cotinine (left) and 3HC (right) concentrations by two questions: “How many cigarettes (on average) are smoked at the home daily in the child’s early life”, and “How many smokers lived at the home during pregnancy?”. Vertical lines reflect cut-points of assumed exposure to very little to no SHS or THS (left), moderate SHS (middle), and regular SHS exposure (right). The dashed lines reflect 0.25 and 30 ng/mL, while dotted lines indicate 0.50 and 60 ng/mL. Density curves were not created for categories with two or fewer participants.

After correcting for urine dilution and imputing those below detection (Supplementary Fig. 3a, b and Supplementary Table 3), the geometric mean cotinine concentration was 0.12 ng/mL (95% CI: 0.11–0.13), and the geometric mean 3HC concentration was 0.22 ng/mL (95% CI: 0.21–0.24) (Table 2). Without correction for dilution, the geometric mean concentrations would be slightly higher at 0.13 ng/mL (95% CI: 0.12–0.14) for cotinine and 0.25 ng/mL (95% CI: 0.21–0.24) for 3HC. The arithmetic mean (and median) concentrations for cotinine were 1.87 ng/mL (median 0.08 ng/mL), and 6.67 ng/mL (median 0.16 ng/mL) for 3HC (Table 2). Based on geometric mean concentrations and density plots (Fig. 1), those who reported no known exposure to tobacco smoke had a geometric mean urinary cotinine concentration of 0.09–0.12 ng/mL, while those who reported some exposure had a more variable range, and those with reported SHS exposure had an average cotinine concentration of at least 0.25 ng/mL depending on the characterizing question.

**Table 2:** Summary statistics of each metabolite.

Metabolite	10th %	25th %	Median	Mean	75th %	90th %	SD	Geometric mean (95% CI)
Cotinine^a^	0.02	0.04	0.08	1.87	0.23	0.77	13.73	0.12 (0.11–0.13)
3HC^a^	0.04	0.07	0.16	6.67	0.45	1.90	67.84	0.22 (0.21–0.24)

*SD* standard deviation, % percentile of distribution range.

^a^Corrected for specific gravity and with concentrations imputed below the level of detection. Cotinine and 3HC are measured in units of ng/mL.

### Concentration cut-points

Applying cut-points of presumed exposure to our samples, based on threshold identified by Benowitz et al. for a population of adolescents with low-smoking exposure [32], we suspect that 1.6% (*n* = 33) of our sample were actively exposed or exposed to recent heavy SHS (≥30 ng/mL), 21.9% (*n* = 441) were exposed to moderate SHS (0.25–30 ng/mL), and 76.5% (*n* = 1543) were exposed to low second or THS to none at all (<0.25 ng/mL). The same concentration thresholds did not apply well to 3HC concentrations, so cotinine thresholds of 0.25 and 30 ng/mL were doubled. When the data are visualized in density curves, the cut-point line would need to be shifted for the 3HC concentrations to match the location on the curve for the cut-point on the cotinine concentration density curves, based upon key questionnaire responses (Fig. 1). Our proposed cut-point values are based on corrected concentrations, and uncorrected concentrations would require a slightly higher cut-point to similarly differentiate infants with suspected tobacco smoke exposure. For uncorrected cotinine, this would mean using 0.31 and 37.6 ng/mL instead of 0.25 and 3 ng/mL. For uncorrected 3HC, cut-points would be approximately 0.63 and 75 ng/mL instead of 0.50 and 60.0 ng/mL.

### Prediction models

**Fig. 2:**
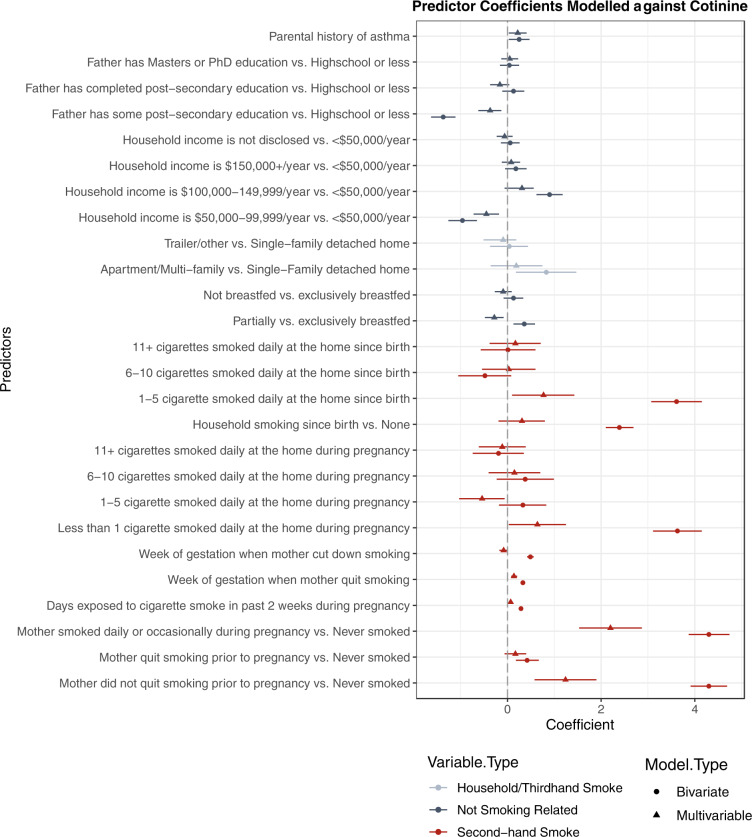
Cotinine multivariable linear regression model. Standard regression coefficients (point) and their 95% confidence intervals (line) are displayed for each variable in a model predicting log-transformed urinary cotinine concentration. Variables related to secondhand smoke are shown in red, variables not related to smoking in blue, and variables related to household characteristics in gray. Note that the ingestion of breast milk is an indirect source of nicotine from secondhand or thirdhand smoke, or dietary nicotine sources. Intervals with a point estimate of the regression coefficients that are displayed as a circle are based on bivariate analysis between each predictor and urinary cotinine, while estimates displayed with a triangle reflect estimates from a multivariable model.

**Fig. 3:**
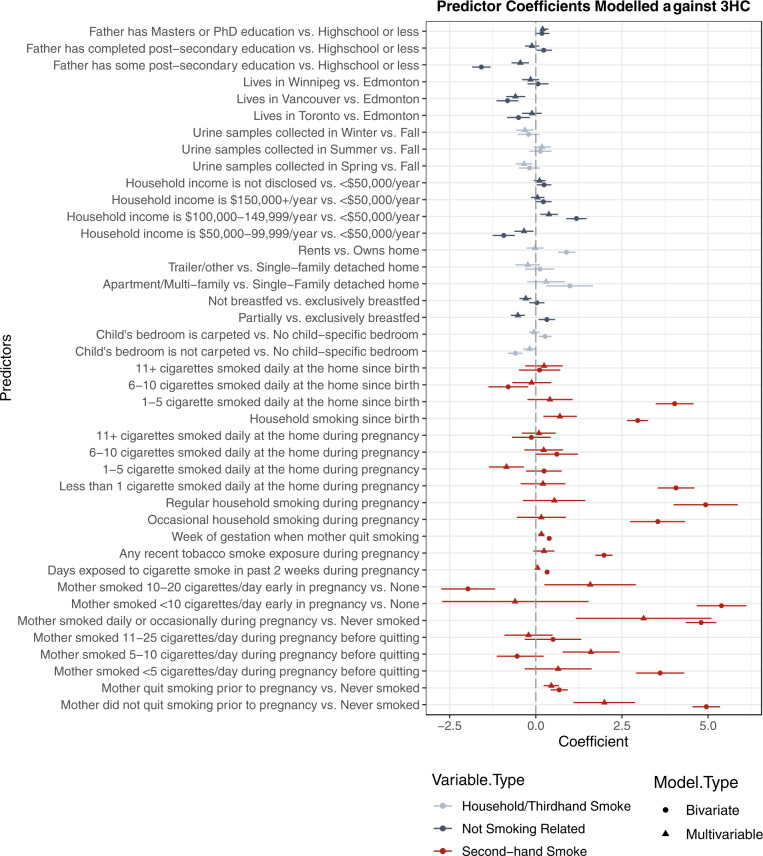
*Trans*-3’-hydroxycotinine multivariable linear regression model. Standard regression coefficients (point) and their 95% confidence intervals (line) are displayed for each variable in a model predicting log-transformed urinary *trans*-3’-hydroxycotinine concentration. Variables related to secondhand smoke are shown in red, variables not related to smoking in blue, and variables related to household characteristics in gray. Note that the ingestion of breast milk is an indirect source of nicotine from secondhand or thirdhand smoke, or dietary nicotine sources. Intervals with a point estimate of the regression coefficients that are displayed as a circle are based on bivariate analysis between each predictor and urinary *trans*-3’-hydroxycotinine concentration while estimates displayed with a triangle reflect estimates from a multivariable model.

**Fig. 4:**
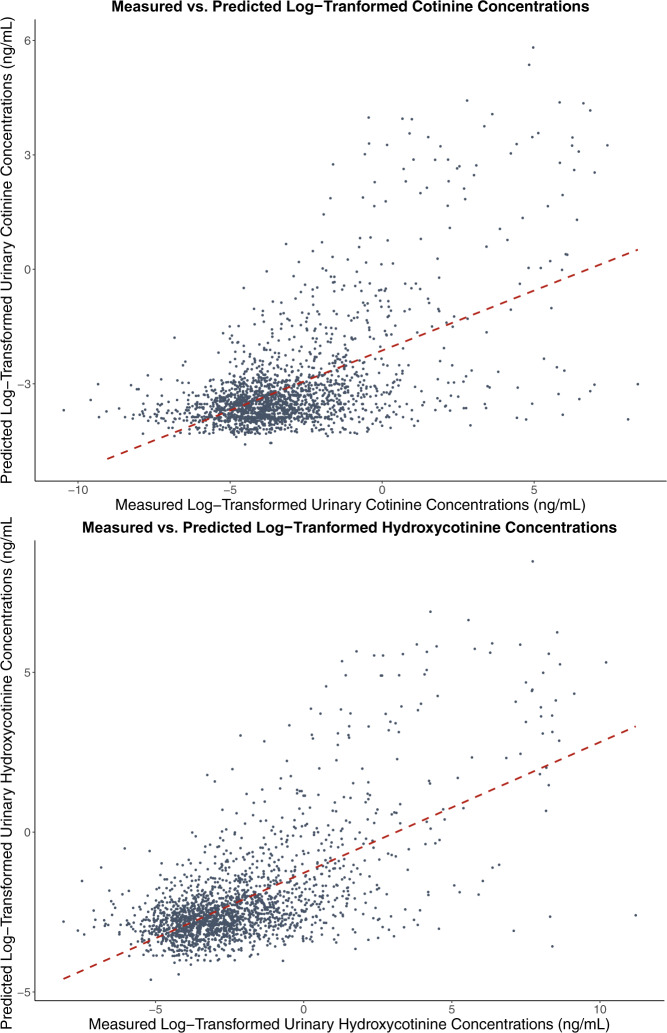
Measured vs. predicted log-transformed urinary cotinine concentration. Scatterplots show the relationship between predicted and measured log-transformed urinary concentrations of cotinine (top) and *trans*-3’-hydroxycotinine (bottom) based on their MLR models (red, dashed line).

The final model selected for cotinine (Fig. 2) used fewer predictors than the final 3HC model (Fig. 3). The selected MLR models predicted 31% of the log-transformed cotinine concentration using 13 predictors and 41% of the variation in the log-transformed 3HC concentration using 19 predictors (Fig. 4). 3HC may require more predictors because its higher concentrations are more sensitive to a breadth of exposure sources. Ten-fold CV found that the models performed slightly poorer, at 30% and 37%, respectively. The most important predictors were whether or not the mother smoked and/or quit prior to the pregnancy, the number of cigarettes smoked at the home during the pregnancy, and whether someone had smoked at the home since the child’s birth, though all predictors added value to the model. Adjusted model coefficients show that those who had not quit prior to their pregnancy had an infant with twice the urinary cotinine concentration compared with an infant of a mother who reportedly never smoked, and mothers actively smoking during their pregnancy had an infant with more than five times the urinary cotinine concentration of infants of non-smoking mothers. A more detailed description of the predictor variables as well as the regression model coefficients and their multiplicative change equivalents can be found in the Supplementary information (Supplementary Tables 4 and 5a, b and Supplementary Fig. 4a, b).

## Discussion

### Urinary nicotine metabolite concentrations

Nicotine exposure was nearly ubiquitous in our study population, with nearly 90% of the urine samples having some detectable level of nicotine metabolite(s). This is similar to a Korean cohort study in which 88% of infants from non-smoking homes had infants with detectable concentrations of cotinine [33] and a study of American adolescents that found that nearly all participants were exposed to tobacco smoke but that the majority of exposure was from light SHS and THS exposure sources [32]. Compared to similar studies [32, 34–36], the urinary concentrations of nicotine metabolites in our study sample were reflective of those with low SHS and/or THS exposure.

3HC had a larger interquartile range (0.38 vs. 0.19 ng/mL) than cotinine, indicating that it varies more in the population and may be a more sensitive biomarker of nicotine exposure. On average, the 3HC concentrations were at least twice that of the cotinine concentrations. Some have proposed that this is due to the difference in the metabolism of nicotine and cotinine between infants and adults. Neonates and children under 1 year of age have lower nicotine metabolism rates, with a nicotine half-life three to four times longer than adults, but the metabolism of cotinine into 3HC in neonates is similar to that of older children and adults [37, 38]. Part of this difference may be due to initially low hepatic blood flow in the transition away from umbilical flow [39], and the lower metabolic enzyme activity for nicotine in early life [40].

### Prediction models

Our study found that prediction models explained less than half of the variation in urinary biomarker concentrations. Overall, approximately half of the predictors included in our models pertained directly to reported SHS exposure (Figs 2 and 3). The predictive multiple linear regression models explained 31.4% of log-transformed cotinine, and 40.9% of 3HC concentrations in our cohort. This prediction is consistent but slightly lower than was reported by other studies of urinary cotinine (33–45%) in children with at least one household smoker that differs from the CHILD cohort [41, 42]. Overall, questionnaire-based models similarly predict biomarker levels across a range of exposure patterns. Parental reports are helpful in characterizing smoke exposure, but less helpful when completed by non-smoking mothers [36]. Others have had more success in predicting serum cotinine (*R*^2^ of 61%) when including indoor air nicotine levels, duration of exposure and ventilation measures [43]. Plotted measured vs. predicted concentrations show some fanning of the observations, particularly at higher concentrations suggesting that the residuals have non-constant variance and that confidence intervals and significance tests should be interpreted with caution (Fig. 4). While our models show how much we could reasonably predict of our biomarker concentrations, they may not be the most parsimonious models. The first two predictors added to the model explained 23% of the log-transformed cotinine concentration, and subsequently added predictors adding between 0.5 and 2% to the *R*^2^ while meeting our inclusion criteria. Some may argue that a model with just these predictors would be a sufficient balance between predictability and the number of predictors required.

Our models are not more predictive of the variation in cotinine concentrations for a few reasons. The half-life of cotinine leads to higher variability for those with low or inconsistent exposure [44]. Another reason may be that our questionnaires are subject to reporting bias and do not adequately detect all aspects of smoke exposure, namely THS exposure. Finally, low but detectable levels of cotinine and 3HC may result from nicotine exposure not related to tobacco smoke, such as diet [45].

While cotinine concentrations remain a reliable way of verifying questionnaire reported tobacco smoking exposure in populations with some level of SHS, they alone may not adequately reflect true exposure in populations with little direct exposure. Prediction models created to explain small-for-gestation size in a Chinese cohort of pregnant women found that self-reported smoking better predicted small-for-gestational-age than cotinine measures [46]. As researchers report lower levels of detection, nicotine exposure in many populations becomes nearly ubiquitous and may begin to lose its predictive power as a proxy of health risks linked to tobacco smoke exposure.

The model to predict 3HC contained more variables, with some of these additional variables related to carpeting, home ownership, and the season at the time of sample collection. More influence due to suspected thirdhand tobacco smoke reservoirs than was found in the cotinine model may exist because 3HC may be a more sensitive marker for chronic, low-level exposure sources. Important predictors to both models include tobacco smoke exposure to the mother during pregnancy and the occurrence of household smoking since the child’s birth. Dwelling type, paternal education, income, and breastfeeding status were used in predicting both urinary cotinine and 3HC. Breastfeeding was likely a surrogate for other characteristics related to tobacco exposure, explaining why those who were breastfed the least had the highest concentrations of urinary cotinine and 3HC before adjusting for smoking factors. Multi-unit or lower-income housing is also more likely to experience THS from previous inhabitants, and SHS from neighboring units [47].

In a prediction model where collinearity and correlated predictors were present, the coefficients became less reliably interpretable. Sparse data bias likely plays a role, a problem where coefficients for levels of a variable with relatively few participants are easily skewed. For these reasons, assessing the geometric mean concentrations by our predictors becomes more helpful than consideration of the adjusted model coefficients.

### Incorporation of machine learning

Random forest analysis identified exposure items relating to breastfeeding, thirdhand sources of tobacco smoke (e.g., carpeting, area rugs), and household characteristics (e.g., single-family detached vs. shared buildings) as being particularly important in predicting the nicotine metabolite concentrations. The advantages of using machine learning methods in environmental epidemiology were recently described as an intelligent way to assess a magnitude of potential exposures and pathways [48].

### Secondhand smoke

Prenatal smoking exposure and related behaviors were important predictors of early life cotinine and 3HC concentrations. Approximately 92% of mothers reported that they never smoked when they became pregnant. Half of the remaining sample of mothers quit during pregnancy. However, one-fifth of mothers reported some recent exposure to tobacco smoke during their pregnancy, a proportion closer to the 24% of mothers who had smoked at some point before or during their pregnancy. Only 7.4% of the participants reported some tobacco smoke to their child at 3–4 months, and 4.4% of the sample reported that the baby had been exposed to tobacco smoke in the past week. By comparison, the 2009 Canadian Maternity Experiences Survey reported that 22% of women smoking daily or occasionally before pregnancy and this proportion reduced to 10.5% during the last 3 months of pregnancy [20]. Of the 183 parents that reported cigarettes were smoked at the home daily in the child’s early life, 62% reported that consumption averaged between 1 and 5 cigarettes per day.

In households where smoking was reported, it was predominantly reported to occur outdoors. This behavior limits the extent and proximity of smoking to the child, and so reflects that parents have some understanding of the dangers of smoking indoors or around an infant. While 12% of our sample reported that someone had smoked at the home since the child’s birth, 11% of the sample reported that smoking occurred outside of the house, and 1.6% reported that smoking occurs near a window or in the garage. Only 0.5% reported smoking occurred inside the home. Although the proportion of smokers in the home was similar before and after birth, the location of where the smoking occurred changed slightly suggesting that avoidance behavior may be greater in the presence of a child than a pregnant woman. While the motivations behind and effects of primarily outdoor smoking are encouraging, smoking outdoors was still associated with approximately three times the urinary cotinine concentrations in infants compared to infants who lived in a home with no household smoking. This suggests the pervasiveness of SHS that overrides efforts to minimize exposure by smoking outdoors.

### Socioeconomic factors

Household income, education level, and maternal age were inversely related to the child’s urinary concentrations. Younger mothers tend to have less formal education, and are less successful in quitting smoking [19]. The dwelling type of the home was important in both prediction models. Those living in apartments or multi-family homes had higher urinary concentration of cotinine and 3HC. SHS is a prominent issue for those in multi-family and multi-unit housing [49]. Children living in rented homes (23%) had higher concentrations of urinary cotinine and 3HC than those who owned their home. Income, education, and housing are all interrelated factors that influence the likelihood of a child being exposed to tobacco smoke in their early life. The inclusion of these variables in prediction models are expected to add value because they capture smoke exposure not already captured by the questions directly related to SHS exposure. We hypothesize that younger, less educated and lower-income mothers may be more likely to have visiting friends and family who smoke or visit public areas where smoking occurs, which may not be reflected in SHS-related responses to our questionnaires.

### Thirdhand smoke

THS is a relatively novel concept, with the term first coined in the late 2000s [50]. THS occurs when SHS interacts with the physical environment and is heavily adsorbed onto surfaces and accumulates in dust [49]. This contamination of surfaces and fabrics from SHS persists to be later released into the air. THS, or residual tobacco smoke pollutants, can be re-emitted as a gas or can react with environmental oxidants or other pollutants to create secondary exposures [6, 49]. Predictors related to household reservoirs of THS, such as carpeting and furnishings, were important in modeling 3HC, though not cotinine. Carpeted flooring and area rugs in the home have been shown to harbor tobacco combustion products [51]. THS exposure can remain elevated for 6 months after smoking cessation, depending on a number of reservoirs such as fabrics, carpets, and dust in the home [52, 53]. Upholstery, carpets, and other fabrics absorb the smoke more readily than other surfaces and can off-gas their contaminants over longer periods of time [52, 54, 55]. Therefore, even if a mother quit smoking prior to her pregnancy, the home may have residual exposure well into her pregnancy.

Characterizing and capturing THS remains a challenge to assessing tobacco smoke exposure. Questionnaires may not accurately capture the complex chemistry of combustion, furnishings, ventilation and human behavior. Thirdhand exposure raises significant challenges for policy makers given the lack of human studies that consider this exposure [6, 49]. Only in the past few years have researchers begun to tease out the effects and pervasiveness of THS [54, 56–58]. As a relatively new phenomena in public health, the public lacks awareness and understanding of THS, which may be an important component of tobacco control [59].

There appears to be a lack of understanding of the far-reaching effects of SHS and THS. Some researchers have proposed that nearly 85% of tobacco smoke is invisible [55]. While it may not be odorous or visible, these light exposures still carry risks [60], thereby posing a challenge in knowledge translation and implementation of controls. Although 12% of the sample reported that someone had smoked at the home, only 7.4% reported that any child had any exposure to smoking in early life, illustrating a gap in understanding. Only a quarter of those who reported a household smoker also reported that their baby had some level of smoking exposure. These inconsistencies may be due to social desirability bias, or the lack of awareness of the pervasiveness of SHS and THS exposure. For example, it likely is not widely understood that smoke particles settle onto dust or fabric surfaces, and that day to day activities like crawling can later facilitate THS exposure to an infant.

### Quantifiably characterizing exposure

Density plots (Fig. 1) show that cut-points in urinary concentrations meant to characterize exposure are not perfect reflections of true exposure. Similar to the findings of Dostal et al. [31], there was notable overlap in the distribution of the infants’ urinary concentrations by predictors meant to characterizing them as exposed or not. This makes the recommendation of cut-points for this population more difficult. We cautiously agree with the continued use of 0.25 ng/mL of corrected cotinine concentration as a cut-point to differentiate those from some confirmed moderate SHS to those exposed to more intermittent or THS sources. For 3HC, we recommend doubling the concentration used as cut-point in the distribution of cotinine. The use of the metabolites to characterize tobacco smoke exposure in infants of a population with relatively low exposure is challenged by the natural variability that comes with intermittent exposure, the half-life of these metabolites, and the potential for nicotine to be sourced from diet [32, 61] as well as tobacco smoke.

### Limitations

We acknowledge that the participants in our study may not reflect the vulnerable population most at-risk for tobacco smoke exposure. While the low prevalence of tobacco smoke exposure in this cohort should be celebrated, this cohort may underestimate true exposure experienced by the Canadian population. Cohort studies like the CHILD Study are also generally more affluent, educated, and predisposed to the diseases under study (asthma and allergies) when compared to the general population. Mothers who smoke during pregnancy are more likely to be low socioeconomic status, non-immigrants, single, have a chronic disease, be without a family doctor, and parenting without having attended prenatal classes [12, 62]. As a result, our recommendations from this study can only be applied to populations similar to our cohort and may not be suitable for individuals from demographics linked to higher cigarette use. At the time of data collection of questionnaire responses used by this study, e-cigarette use was not yet popularized, and marijuana use was still illegal in Canada. We also did not have measure of the maternal use of nicotine-supplementing smoking cessation products, which may contribute to an infant’s nicotine intake through breastfeeding but not be reflective of inhaled smoke. We recognize that we have been unable to assess for more novel means of tobacco smoke exposure and encourage that future analysis make use of data on marijuana and e-cigarette use when characterizing exposure. As well, we were unable to consider variability in CYP2A6 enzyme function in participants, which could affect nicotine metabolism and clearance.

## Conclusion and future directions

While our smoking rates were low and parents appeared to be motivated to avoid exposing the child to smoke, nicotine metabolites were nearly ubiquitous in infants’ urine. Our results suggest that tobacco smoke questionnaire models may not accurately explain the majority of variation in cotinine or 3HC concentrations within a population with relatively little smoking exposure. Questions that best explained the variation in nicotine metabolite concentrations included whether the mother quit smoking prior to pregnancy, the number of cigarettes smoked daily at the home during pregnancy, and the presence of household smoking since the child’s birth. Tobacco smoke exposure models could use a combination of questionnaire and biomarker data to more accurately assess risk or consider additional exposure assessment tools like air sampling, dust and surface wipe sample collection. Future models should also consider  to what extent smoking is occurring at work, hospitality venues, parks, preschools and other places where families with young children gather. As the Canadian population overall is reducing the rates of smoking, particularly during pregnancy and when around children—both as a result of policy and social pressures—our understanding of cotinine as a measure of smoking needs to change. As smoking rates drop, low-level concentrations may be influenced by thirdhand, dietary, and therapeutic sources of nicotine. Researchers need to be aware of the context of their sample population and be purposeful in the selection of the appropriate exposure measures.

With growing public health concern over e-cigarettes and marijuana smoking, this study highlights a lack of awareness of light SHS and THS as an additional frontier of tobacco smoke research and action. Physicians and care providers are encouraged to have conversations with their patients and their families about SHS and THS exposure and possible remedies. This study encourages researchers to look for better means of exposure measurement in populations with relatively low or intermittent tobacco smoking. Future work should focus on collaboration between qualitative and quantitative analysis to better understand the motivations behind smoking cessation or reducing exposure to children, and how the implications of SHS are understood by the public. The pervasiveness of THS and any nicotine ingested through dust or breast milk not necessarily from smoke exposure should be investigated in populations with low reported exposure.

## Supplementary information

Supplementary information
