# Supplementary material for: Assessing secondhand and thirdhand tobacco smoke exposure in Canadian infants using questionnaires, biomarkers, and machine learning
Source: J Expo Sci Environ Epidemiol. 2021 Jun 26;32(1):112–23. doi: 10.1038/s41370-021-00350-4 (PMC8770125; doi:10.1038/s41370-021-00350-4)
Supplement: Supplementary file 1 — Supplementary information [file 41370_2021_350_MOESM1_ESM.docx]

**Appendix to *Assessing secondhand and thirdhand tobacco smoke exposure in Canadian infants using questionnaires, biomarkers, and machine learning***

**Table of Contents**

[**Figure 1:** Sample selection flow chart (n=2,017)](#F1)

[**Table 1a:** Distribution of metabolite concentrations by smoking exposure](#T1a)

[**Table 1b:** Distribution of metabolite concentrations by household characteristics](#T1b)

[**Table 1c:** Distribution of metabolite concentrations by socioeconomic factors, parental disease history and ethnicity, and infant diet.](#T1c)

[**Figure 2a:** Variable importance plot for cotinine concentration](#F2a)

[**Figure 2b:** Variable importance plot for *trans*-3’-Hydroxycotinine concentration](#F2b)

[**Table 2:**](#T2) Variable importance and R^2^ scores for potential predictors

[**Figure 3a:** Distribution of log-transformed Cotinine concentration following imputation of concentrations below detection](#F3a)

[**Figure 3b:** Distribution of log-transformed *trans*-3’-Hydroxycotinine concentration following imputation of concentrations below detection](#F3b)

[**Table 3**: Summary statistics of each metabolite with and without dilution correction](#T3)

[**Table 4:** Predictor data dictionary](#T4)

[**Table 5a**](#T5a)**:** Cotinine Multivariable Linear Regression Model and Multiplicative Change in log-transformed Cotinine concentrations

[**Table 5b**](#T5b)**:** *Trans*-3’-Hydroxycotinine Multivariable Linear Regression Model and Multiplicative Change in log-transformed *trans*-3’-Hydroxycotinine concentrations

[**Figure 4a:**](#F4a) Multiplicative Change in Cotinine Multivariable Linear Regression Model

[**Figure 4b:**](#F4b) Multiplicative Change in *trans*-3’-Hydroxycotinine Multivariable Linear Regression Model

**Figure 1. Sample Selection Flow Chart.**

Of the 3,455 children in the CHILD Study, 2,607 had urine samples collected at the 3-4 month home visit. 2,607 urine samples are available, with 2,509 of these samples remaining after excluding those with inconclusive results errors. These results errors include results that were duplicates, experienced interference with the sample, had insufficient volume for testing, or with a specific gravity measures above a 3 standard deviation cutoff. Of the 2,509 participants with metabolite data, 589 (23.5%) had a cotinine concentration below the LOD, and 271 (10.8%) had a 3HC concentration below the LOD. A subset of these 2,509 who have complete data, including those who could not have a result reported because the concentration was below the level of detection (0.03ng/mL) were selected as the current sample size. This sample was then restricted to those who also had complete information for all demographic and potential predictors of tobacco smoke exposure, limiting the sample size to 2,017.

**Table 1a:** Distribution of Metabolite Concentrations by Smoking Exposure

| **Predictor Variable** | **% (N)** | **Geometric mean urinary Cotinine (95% CI), ng/mL** | **Geometric mean urinary Hydroxycotinine (95% CI), ng/mL** |
| --- | --- | --- | --- |
| **Prenatal maternal smoking** | | **p<0.001** | **p<0.001** |
| Never Smoked | 91.9% (n=1853) | 0.10 (0.09-0.10) | 0.18 (0.17-0.19) |
| Quit during pregnancy | 4.4% (n=88) | 0.82 (0.52-1.29) | 2.30 (1.34-3.93) |
| Did not quit smoking | 3.9% (n=76) | 1.15 (0.62-2.16) | 3.24 (1.68-6.26) |
| **Maternal smoking status in pregnancy** | | **p<0.001** | **p<0.001** |
| Never Smoked | 97.5% (n=1967) | 0.10 (0.10-0.11) | 0.20 (0.19-0.21) |
| Daily of Occasional Smoker | 2.6% (n=50) | 7.13 (4.18-12.14) | 21.96 (12.21-39.48) |
| **Pre-prenatal maternal smoking** | | **p<0.001** | **p<0.001** |
| Never Smoked | 76.1% (n=1535) | 0.09 (0.09-0.10) | 0.17 (0.16-0.18) |
| Quit prior to pregnancy | 17.7% (n=358) | 0.12 (0.10-0.15) | 0.27 (0.23-0.31) |
| Did not quit prior to pregnancy | 6.1% (n=124) | 1.81 (1.19-2.77) | 5.16 (3.19-8.34) |
| **Was the mother exposed to a smoker at the home during pregnancy** | | **p<0.001** | **p<0.001** |
| Yes, Regular Smoker | 1.2% (n=25) | 2.04 (0.97-4.32) | 6.26 (2.87-13.69) |
| Yes, Occasional Smoker | 1.7% (n=35) | 0.85 (0.44-1.62) | 2.39 (1.16-4.92) |
| No | 97.0% (n=1957) | 0.11 (0.10-0.12) | 0.21 (0.19-0.22) |
| **Smoker lives at the home during pregnancy** | | **p<0.0001** | **p<0.0001** |
| No smoker at the home | 88.9% (n=1794) | 0.10 (0.09-0.10) | 0.18 (0.17-0.19) |
| Yes, smoker at the home | 11.1% (n=223) | 0.56 (0.44-0.71) | 1.44 (1.11-1.87) |
| **Where household smoking occurs during pregnancy** | | **p<0.0001** | **p<0.0001** |
| Inside | 1.0% (n=20) | 3.00 (1.46-6.15) | 8.58 (3.68-19.99) |
| Near a Window or in Garage | 1.9% (n=38) | 0.91 (0.54-1.55) | 2.62 (1.39-4.94) |
| Outside | 8.8% (n=178) | 0.47 (0.36-0.61) | 1.19 (0.89-1.59) |
| **Mother reports exposure during pregnancy** | | **p<0.0001** | **p<0.0001** |
| Recent Exposure | 21.1% (n=425) | 0.29 (0.24-0.35) | 0.66 (0.54-0.81) |
| No recent exposures | 78.9% (n=1592) | 0.09 (0.08-0.10) | 0.29 (0.24-0.35) |
| **Mother reports average daily exposure to smoke during pregnancy (continuous)** | | **p <0.0001** | **p<0.0001** |
| None | 79.0% (n=1593) | 0.09 (0.08-0.10) | 0.17 (0.16-0.26) |
| 0.25 hr/day | 14.8% (n=299) | 0.21 (0.18-0.26) | 0.46 (0.38-0.57) |
| 0.5-1 hr/day | 3.6% (n=72) | 0.49 (0.27-0.89) | 1.10 (0.57-2.13) |
| 2-4hr/day | 1.4% (n=29) | 0.45 (0.19-1.07) | 1.18 (0.46-3.02) |
| 5-24hr/day | 1.2% (n=24)) | 1.91 (0.72-5.09) | 6.05 (2.16-16.92) |
| **Mothers days exposure to smoke during pregnancy in past 2 weeks (continuous)** | | **p<0.0001** | **p<0.0001** |
| None | 78.9% (n=1592) | 0.09 (0.08-0.10) | 0.17 (0.16-0.18) |
| 1 day | 5.0% (n=100) | 0.12 (0.09-0.16) | 0.26 (0.19-0.35) |
| 2 days | 4.1% (n=83) | 0.17 (0.12-0.23) | 0.39 (0.28-0.54) |
| 3-4 days | 4.0% (n=80) | 0.22 (0.15-0.32) | 0.45 (0.30-0.69) |
| 5-6 days | 1.2% (n=24) | 0.27 (0.14-0.51) | 0.50 (0.27-0.94) |
| 7-8 day | 1.3% (n=27) | 0.26 (0.15-0.47) | 0.45 (0.22-0.94) |
| 9-12 days | 1.3% (n=27) | 0.38 (0.18-0.80) | 1.08 (0.46-2.55) |
| 14 days (every day) | 4.2% (n=84) | 1.84 (1.09-3.10) | 5.09 (2.87-9.03) |
| **Someone has smoked at the home since birth** | | **p<0.0001** | **p<0.0001** |
| No smoking at the home | 87.8% (n=1771) | 0.10 (0.09-0.10) | 0.17 (0.16-0.19) |
| Yes, smoking at the home | 12.2% (n=246) | 0.50 (0.38-0.65) | 1.36 (1.02-1.81) |
| **Where household smoking occurs during child’s early life** | | **Inside v. None p<0.001**  **Window v. None p<0.001**  **Outside v. None p<0.001** | **Inside v. None p<0.001**  **Window v. None p<0.001**  **Outside v. None p<0.001** |
| Inside | 0.5% (n=10) | 2.07 (0.79-5.44) | 6.45 (2.50-16.63) |
| Near a Window or in Garage | 1.6% (n=32) | 1.30 (0.63-2.71) | 4.18 (1.98-8.85) |
| Outside | 11.2% (n=225) | 0.43 (0.33-0.57) | 1.16 (0.86-1.57) |
| **Cigarettes smoked daily at the home during child’s early life** | | **p<0.0001** | **p<0.0001** |
| None | 91.0% (n=1836) | 0.10 (0.09-0.10) | 0.18 (0.17-0.19) |
| 1-5 cigarettes/day | 5.6% (n=113) | 0.42 (0.29-0.60) | 1.17 (0.78-1.74) |
| 6-10 cigarettes/day | 1.7% (n=35) | 1.27 (0.64-2.50) | 3.69 (1.74-7.82) |
| 10+ cigarettes/day | 1.6% (n=33) | 2.80 (1.29-6.08) | 7.93 (3.37-18.63) |
| **Parent reports smoking exposure to child in early life** | | **p<0.0001** | **p<0.0001** |
| None | 92.6% (n=1868) | 0.11 (0.10-0.12) | 0.20 (0.19-0.22) |
| Some | 7.4% (n=149) | 0.30 (0.22-0.40) | 0.80 (0.59-1.09) |
| **Days baby was exposed to smoke during the past week, early life (continuous)** | | **p<0.0001** | **p<0.0001** |
| None | 95.6% (n=1928) | 0.11 (0.10-0.12) | 0.21 (0.19-0.22) |
| 1 day | 2.1% (n=43) | 0.33 (0.20-0.54) | 0.78 (0.47-1.30) |
| 2 days | 0.7% (n=14) | 0.26 (0.10-0.71) | 0.96 (0.33-2.82) |
| 3-5 days | 0.5% (n=9) | 0.19 (0.06-0.62) | 0.50 (0.13-2.02) |
| 6-7 days | 1.1% (n=23) | 1.30 (0.46-3.64) | 4.11 (1.55-10.91) |
| **Hours of smoke exposure in the past week to child, early life** | | **p<0.0001** | **p<0.0001** |
| None | 95.6% (n=1929) | 0.11 (0.10-0.12) | 0.21 (0.19-0.22) |
| 10 hours /week | 3.7% (n=74) | 0.37 (0.24-0.58) | 1.02 (0.64-1.61) |
| 10 to 20 hours/week | 0.2% (n=5) | 0.78 (0.10-6.34) | 1.60 (0.14-18.76) |
| More than 20 hours/week | 1.0% (n=20) | 1.20 (0.29-5.04) | 4.01 (1.01-15.85) |

The proportion and crude number of sample subjects that corresponds to each level of household characteristic variables is reported to the nearest whole number. The geometric mean (95% Confidence interval) of the corrected and log-transformed Cotinine distribution for each level of each variable is also reported to the nearest two decimal places. P-values represent test for comparing means between levels of each predictor. P-values less than 0.05 are bolded.

**Table 1b:** Distribution of Metabolite Concentrations by Household Characteristics

| **Characteristic** | **% (N)** | **Geometric mean urinary Cotinine (95% CI), ng/mL** | | **Geometric mean urinary Hydroxycotinine (95% CI), ng/mL** |
| --- | --- | --- | --- | --- |
| **Dwelling Type** | | | **p<0.0001** | **p<0.0001** |
| Single Family | 55.8% (n=1470) | 0.10 (0.10-0.11) | | 0.20 (0.18-0.22) |
| Multi-Family or Apartment | 25.8% (n=521) | 0.15 (0.13-0.18) | | 0.29 (0.25-0.34) |
| Trailer or Other | 1.2% (n=26) | 0.24 (0.11-0.49) | | 0.53 (0.23-1.19) |
| **Carpeted Flooring** | | | CLS v. Not p=0.06  CMR v. Not p=0.07  **CCR** v. Not **p<0.0001** | CLS v. Not p=0. 19  **CMR** v. Not **p=0.007**  **CCR** v. Not **p<0.0001** |
| Carpeted Living Space (LS) | 26.7% (n=539) | 0.13 (0.11-0.15) | | 0.24 (0.20-0.27) |
| Living Space Not Carpeted | 73.3% (n=1478) | 0.11 (0.10-0.12) | | 0.22 (0.20-0.24) |
| Carpeted Mom’s Room (MR) | 50.0% (n=1009) | 0.11 (0.10-0.12) | | 0.21 (0.19-0.23) |
| Mom’s Room not carpeted | 50.0% (n=1008) | 0.12 (0.11-0.13) | | 0.24 (0.22-0.27) |
| Carpeted Child’s Room (CR) | 39.9% (n=804) | 0.10 (0.09-0.12) | | 0.20 (0.18-0.22) |
| Child’s Room Not Carpeted | 42.5% (n=857)  *356 no child’s room | 0.11 (0.10-0.13) | | 0.21 (0.19-0.23) |
| **Air Conditioning (AC)** | | | **p=0.00001** | **p<0.00001** |
| None | 55.3% (n=1115) | 0.12 (0.11-0.13) | | 0.21 (0.18-0.23) |
| Central AC | 49.2% (n=992) | 0.10 (0.09-0.11) | | 0.20 (0.18-0.22) |
| Window or Portable Unit | 19.9% (n=402) | 0.17 (0.14-0.20) | | 0.38 (0.31-0.46) |
| **Rent vs. Own Home** | | | **p<0.0001** | **p<0.0001** |
| Rent | 34.3% (n=692) | 0.19 (0.16-0.22) | | 0.36 (0.31-0.43) |
| Own | 90.1% (n=1817) | 0.10 (0.09-0.11) | | 0.19 (0.18-0.21) |
| **Bedrooms in Home** | | | p=0.09 | **p=0.007** |
| No bedrooms | <1% (n=2) | 0.14 (N/A) | | 0.99 (N/A) |
| 1-3 bedrooms | 73.2% (n=1476) | 0.12 (0.11-0.13) | | 0.24 (0.22-0.26) |
| 4-6 bedrooms | 26.3% (n=531) | 0.10 (0.09-0.12) | | 0.19 (0.16-0.22) |
| 7+ bedrooms | 0.4% (n=8) | 0.08 (0.03-0.22) | | 0.15 (0.05-0.48) |
| **Area Rug locations** | | | **B p=0.003**  K p=0.68  **LS p=0.02**  MB p=0.56  **B p=0.05** | **B p=0.008**  K p=0.18  **LS p=0.003**  MB p=0.08  **B p=0.007** |
| Basement (B) | 31.6% (n=637) | 0.10 (0.09-0.11) | | 0.20 (0.17-0.22) |
| Not in Basement | 37.5% (n=756) | 0.11 (0.10-0.12) | | 0.21 (0.19-0.24) |
| No Basement | 30.9% (n=624) | 0.15 (0.13-0.17) | | 0.27 (0.24-0.31) |
| Kitchen (K) | 18.2% (n=368) | 0.12 (0.10-0.14) | | 0.21 (0.17-0.25) |
| Not in Kitchen | 81.8% (n=1649) | 0.12 (0.11-0.13) | | 0.23 (0.21-0.25) |
| Living Space (LS) | 45.0% (n=907) | 0.11 (0.10-0.12) | | 0.20 (0.18-0.22) |
| Not in Living Space | 55.0% (n=1110) | 0.12 (0.11-0.13) | | 0.24 (0.22-0.27) |
| Mom’s bedroom (MB) | 14.0% (n=282) | 0.11 (0.10-0.13) | | 0.24 (0.20-0.29) |
| Not in Mom’s bedroom | 86.0% (n=1735) | 0.12 (0.11-0.13) | | 0.22 (0.20-0.24) |
| Bathroom (B) | 35.1% (n=707) | 0.11 (0.10-0.12) | | 0.19 (0.17-0.22) |
| Not in Bathroom | 64.9% (n=1310) | 0.12 (0.11-0.13) | | 0.24 (0.22-0.27) |
| **Car Ownership** | | | **p=0.002** | **p=0.00002** |
| No Cars | 6.0% (n=122) | 0.20 (0.14-0.28) | | 0.45 (0.31-0.64) |
| 1 Car | 41.4% (n=835) | 0.12 (0.10-0.13) | | 0.21 (0.19-0.24) |
| 2 Car | 46.7% (n=942) | 0.11 (0.10-0.12) | | 0.21 (0.19-0.23) |
| 3+ Cars | 5.9% (n=118) | 0.11 (0.08-0.15) | | 0.28 (0.20-0.39) |

The proportion and crude number of sample subjects that corresponds to each level of household characteristic variables is reported to the nearest whole number. The geometric mean (95% Confidence interval) of the corrected and log-transformed Cotinine distribution for each level of each variable is also reported to the nearest two decimal places. P-values represent test for comparing means between levels of each predictor. P-values less than 0.05 are bolded. The ‘Area Rug Location’ rows detail whether an area rug was or was not present in the basement, kitchen, living space, or bedrooms. Similarly, for ‘Carpeted Flooring’, the rows indicate whether the flooring in each of the rooms listed was primarily carpeting.

**Table 1c:** Distribution of Metabolite Concentrations by Socioeconomic factors, parental disease history and ethnicity, and infant diet.

| **Characteristic** | **% (N)** | **Geometric mean urinary Cotinine (95% CI), ng/mL** | | **Geometric mean urinary Hydroxycotinine (95% CI), ng/mL** |
| --- | --- | --- | --- | --- |
| **Household Income** | | | **p<0.0001** | **p<0.0001** |
| $0-49,999/year | 10.0% (n=201) | 0.26 (0.20-0.34) | | 0.55 (0.43-0.76) |
| $50,000-99,999/year | 31.3% (n=631) | 0.14 (0.12-0.16) | | 0.26 (0.22-0.29) |
| $100,000-149,999/year | 26.4% (n=533) | 0.09 (0.08-0.11) | | 0.19 (0.16-0.21) |
| $150,000+/year | 23.3% (n=469) | 0.08 (0.07-0.09) | | 0.14 (0.12-0.16) |
| Prefers to not say | 9.1% (n=183) | 0.12 (0.10-0.15) | | 0.28 (0.22-0.36) |
| **Paternal Education** | | | **p<0.0001** | **p<0.0001** |
| Highschool or less | 12.4% (n=250) | 0.26 (0.20-0.33) | | 0.59 (0.45-0.78) |
| Some Post-Secondary | 16.7% (n=336) | 0.16 (0.13-0.20) | | 0.35 (0.28-0.43) |
| Completed Post-Secondary | 54.5% (n=1099) | 0.10 (0.09-0.11) | | 0.18 (0.16-0.20) |
| Masters or PhD | 16.5% (n=332) | 0.07 (0.06-0.09) | | 0.14 (0.13-0.17) |
| **Maternal Education** | | | **p<0.0001** | **p<0.0001** |
| Highschool or less | 6.6% (n=133) | 0.30 (0.21-0.42) | | 0.75 (0.52-1.08) |
| Some Post-Secondary | 14.0% (n=282) | 0.18 (0.14-0.22) | | 0.35 (0.28-0.44) |
| Completed Post-Secondary | 59.3% (n=1196) | 0.10 (0.09-0.11) | | 0.20 (0.18-0.22) |
| Masters or PhD | 20.1% (n=406) | 0.09 (0.08-0.10) | | 0.16 (0.14-0.19) |
| **Parental Medical History** | | | **A vs. no A p=0.03**  ATP vs. no ATP p=0.19 | **A vs. no A** p=0.11  **ATP vs. no ATP p=0.02** |
| Asthma (A) | 31.3% (n=660) | 0.13 (0.12-0.15) | | 0.24 (0.21-0.27) |
| No Asthma | 67.3% (n=1357) | 0.11 (0.10-0.12) | | 0.22 (0.20-0.24) |
| Atopy (ATP) | 79.9% (n=1611) | 0.11 (0.11-0.12) | | 0.22 (0.20-0.23) |
| No Atopy | 20.1% (n=406) | 0.13 (0.11-0.15) | | 0.26 (0.22-0.31) |
| **Month of Birth** | | | p=0.18 | p=0.22 |
| January | 8.4% (n=170) | 0.11 (0.09-0.14) | | 0.20 (0.15-0.25) |
| February | 7.6% (n=153) | 0.14 (0.11-0.18) | | 0.24 (0.18-0.33) |
| March | 9.9% (n=200) | 0.12 (0.10-0.15) | | 0.24 (0.19-0.30) |
| April | 8.9% (n=179) | 0.12 (0.10-0.16) | | 0.26 (0.20-0.33) |
| May | 9.0% (n=182) | 0.12 (0.10-0.15) | | 0.23 (0.18-0.28) |
| June | 9.1% (n=184) | 0.10 (0.08-0.13) | | 0.23 (0.18-0.29) |
| July | 8.2% (n=166) | 0.12 (0.08-0.16) | | 0.27 (0.19-0.36) |
| August | 7.2% (n=145) | 0.12 (0.09-0.15) | | 0.26 (0.19-0.35) |
| September | 6.9% (n=140) | 0.13 (0.09-0.18) | | 0.21 (0.15-0.30) |
| October | 7.7% (n=156) | 0.09 (0.07-0.12) | | 0.16 (0.13-0.19) |
| November | 8.9% (n=179) | 0.12 (0.10-0.15) | | 0.20 (0.16-0.25) |
| December | 8.1% (n=163) | 0.11 (0.08-0.14) | | 0.23 (0.17-0.30) |
| **Season of sample Collection** | | | **p=0.01** | **p=0.0008** |
| Summer | 24.7% (n=498) | 0.13 (0.11-0.15) | | 0.26 (0.22-0.30) |
| Fall | 23.2% (n=467) | 0.11 (0.09-0.13) | | 0.24 (0.20-0.27) |
| Winter | 23.8% (n=481) | 0.11 (0.09-0.13) | | 0.20 (0.17-0.24) |
| Spring | 28.3% (n=571) | 0.12 (0.10-0.13) | | 0.21 (0.18-0.24) |
| **Maternal Ethnicity** | | | **p=0.0001** | **p<0.0001** |
| Caucasian/White | 74.8% (n=1509) | 0.11 (0.11-0.12) | | 0.22 (0.20-0.24) |
| East Asian | 6.3% (n=127) | 0.09 (0.07-0.11) | | 0.13 (0.11-0.17) |
| South East Asian | 4.8% (n=97) | 0.13 (0.10-0.18) | | 0.20 (0.14-0.28) |
| Multiracial | 4.1% (n=82) | 0.10 (0.08-0.15) | | 0.22 (0.15-0.33) |
| First Nations | 3.4% (n=69) | 0.39 (0.23-0.65) | | 1.02 (0.57-1.82) |
| South Asian | 2.6% (n=53) | 0.09 (0.06-0.13) | | 0.22 (0.13-0.34) |
| Hispanic | 1.5% (n=31) | 0.10 (0.06-0.17) | | 0.16 (0.10-0.26) |
| Black | 1.3% (n=27) | 0.12 (0.08-0.18) | | 0.28 (0.17-0.45) |
| Middle Eastern | 0.9% (n=19) | 0.09 (0.05-0.15) | | 0.24 (0.14-0.42) |
| Unknown/Other | 0.1% (n=3) | 0.07 (0.003-1.46) | | 0.21 (0.01-3.49) |
| **Paternal Ethnicity** | | | **p=0.002** | **p<0.0001** |
| Caucasian/White | 75.4% (n=1521) | 0.11 (0.10-0.12) | | 0.21 (0.20-0.23) |
| East Asian | 4.7% (n=95) | 0.09 (0.07-0.12) | | 0.13 (0.10-0.18) |
| South East Asian | 3.9% (n=79) | 0.10 (0.07-0.14) | | 0.16 (0.12-0.22) |
| Multiracial | 3.4% (n=68) | 0.12 (0.08-0.18) | | 0.24 (0.17-0.36) |
| First Nations | 3.7% (n=75) | 0.28 (0.18-0.45) | | 0.65 (0.38-1.09) |
| South Asian | 3.5% (n=70) | 0.11 (0.08-0.15) | | 0.22 (0.15-0.32) |
| Hispanic | 1.5% (n=31) | 0.12 (0.06-0.25) | | 0.23 (0.11-0.48) |
| Black | 2.1% (n=45) | 0.18 (0.09-0.33) | | 0.65 (0.32-1.33) |
| Middle Eastern | 1.0% (n=20) | 0.08 (0.05-0.12) | | 0.19 (0.12-0.30) |
| Unknown/Other | 0.6% (n=13) | 0.25 (0.07-0.85) | | 0.52 (0.12-2.17) |
| **Maternal Age at Enrolment** | | | **p<0.0001** | **p<0.0001** |
| 17 to 23 years old | 3.8% (n=77) | 0.42 (0.28-0.64) | | 1.01 (0.64-1.60) |
| 24 to 30 years old | 32.2% (n=649) | 0.14 (0.12-0.16) | | 0.29 (0.25-0.33) |
| 31 to 35 years old | 41.9% (n=845) | 0.10 (0.09-0.11) | | 0.19 (0.17-0.21) |
| 36-46 years old | 22.1% (n=446) | 0.09 (0.08-0.11) | | 0.17 (0.15-0.20) |
| **Study Centre** | | | **p=0.00005** | **p<0.0001** |
| Vancouver | 26.7% (n=539) | 0.10 (0.09-0.12) | | 0.16 (0.14-0.18) |
| Edmonton | 19.9% (n=402) | 0.13 (0.11-0.15) | | 0.28 (0.23-0.34) |
| Winnipeg (+ nearby rural) | 30.9% (n=624) | 0.14 (0.12-0.16) | | 0.29 (0.25-0.34) |
| Toronto | 22.4% (n=452) | 0.09 (0.08-0.10) | | 0.20 (0.17-0.22) |
| **Breastfeeding Status** | | | **p=0.005** | p=0.16 |
| None | 12.0% (n=243) | 0.16 (0.13-0.19) | | 0.29 (0.23-0.36) |
| Partial | 25.8% (n=520) | 0.12 (0.10-0.14) | | 0.24 (0.20-0.28) |
| Exclusive | 62.2% (n=1254) | 0.11 (0.10-0.12) | | 0.21 (0.19-0.23) |
| **Infant’s 3-month Diet** | | | **p=0.04** | p=0.18 |
| Breastfeeding Only | 62.2% (n=1254) | 0.11 (0.10-0.12) | | 0.21 (0.19-0.23) |
| Breastfeeding and Formula | 24.2% (n=489) | 0.12 (0.10-0.14) | | 0.24 (0.20-0.28) |
| Breastfeeding and Solid Food | 0.9% (n=18) | 0.13 (0.04-0.37) | | 0.34 (0.11-1.02) |
| Formula Only | 11.2% (n=226) | 0.15 (0.12-0.19) | | 0.27 (0.21-0.34) |
| Formula and Solid Food | 0.8% (n=17) | 0.27 (0.09-0.77) | | 0.71 (0.23-2.17) |
| Breastfeeding, Formula, and Solid Food | 0.6% (n=13) | 0.12 (0.05-0.28) | | 0.20 (0.06-0.63) |

The proportion and crude number of sample subjects that corresponds to each level of household characteristic variables is reported to the nearest whole number. The geometric mean (95% Confidence interval) of the corrected and log-transformed Cotinine distribution for each level of each variable is also reported to the nearest two decimal places. P-values represent tests for comparing means between levels of each predictor. P-values less than 0.05 are bolded.


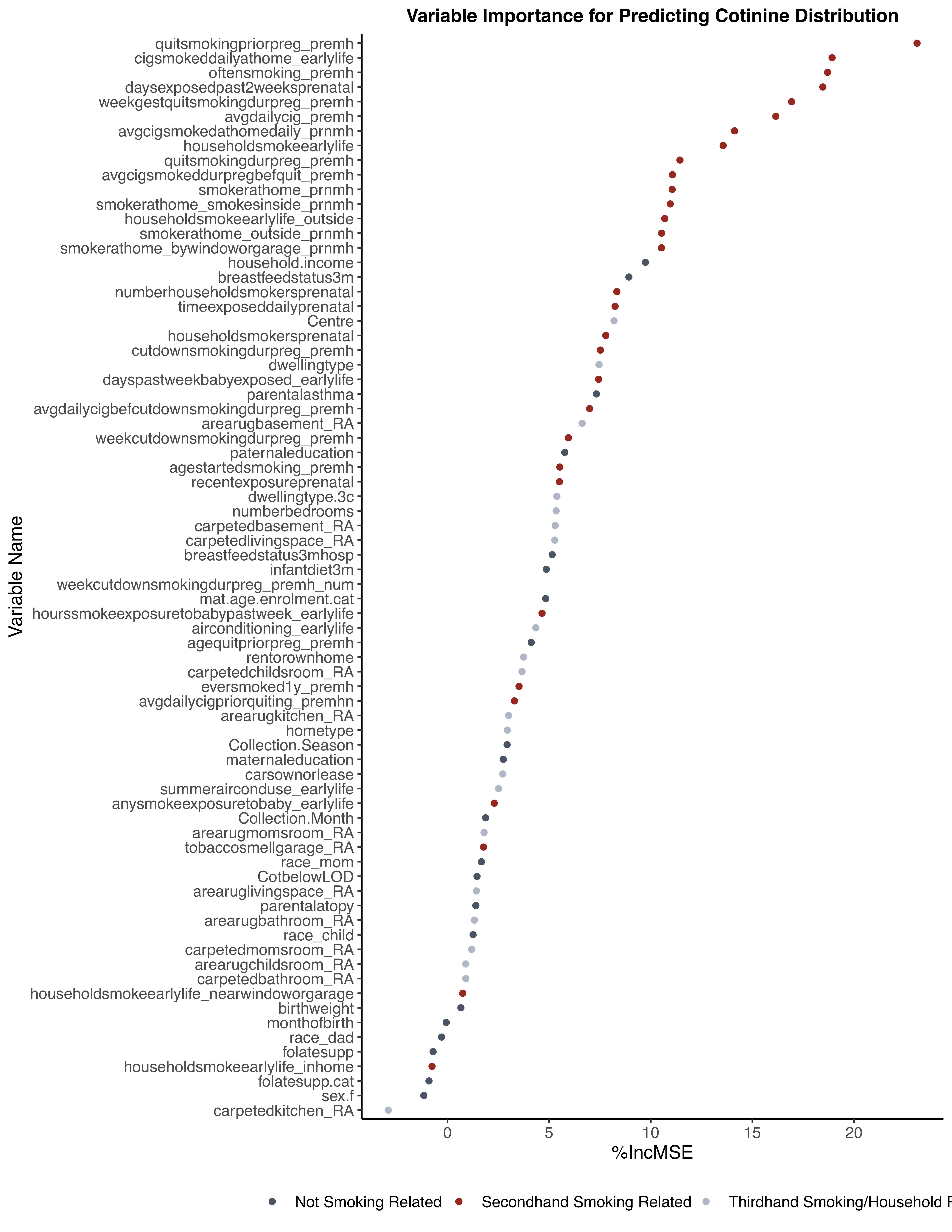


Maternal Smoking status prior to pregnancy
Cigarettes smoked daily at the home, early life

How often did the mother smoke during pregnancy

Days mother exposed to smoke during pregnancy, past 2 wks

Week Gestation that mother quit smoking

Cigarettes smoked daily by mother during pregnancy

Cigarettes smoked daily at the home, during pregnancy

Household smoking in early life

Mother quit smoking during pregnancy

Cigarettes smoked during pregnancy pre-quitting

Household smoking during pregnancy
Household smoker smokes inside during pregnancy

Household smoker smokes outside, early life
Household smoker smokes outside during pregnancy

Household smoking near window/garage during pregnancy

Household Income

Breastfeeding status at 3 months

Number of smokers at the home during pregnancy

Hours pregnant mother is exposed daily

Study Centre

Household smoker present during pregnancy

Mother reduced smoking during pregnancy

Dwelling type, 6 groups

Days infant exposed to smoke in past week, 3mo

Parental history of asthmatic disease

Cigarettes smoked daily prior to reducing, in pregnancy

Basement has area rug

Week Gestation that mother reduced smoking (grouped)

Father’s highest level of education

Age that the mother started smoking

Any recent smoke exposure during pregnancy

Dwelling type, 3 groups

Number of bedrooms in the home

Basement is carpeted

Primary living space is carpeted

Breastfeeding status, including in hospital

Infant diet

Week Gestation that mother reduced smoking(numeric)

Mother’s age at enrolment

Hours infant exposed to smoke in past week, 3mo

Presence and type of air conditioning, early life

Age mother quit smoking prior to pregnancy

Home ownership

Child’s bedroom is carpeted

Has the mother ever smoked for 1+ year?

Cigarettes smoked daily prior to quitting in pregnancy

Kitchen has an area rug

Single or Multi-family home

Season at sample collection

Mother’s highest level of education

Number of cars owned or leased

Air conditioner use during Summer months

Any smoke exposure to baby since birth

Month of sample collection

Mom’s bedroom has an area rug

Tobacco smell detected in garage

Maternal ethnicity

Cotinine concentration was below LOD

Living space has an area rug

Parental atopy status

Bathroom has an area rug

Child’s ethnicity (derived)

Mom’s bedroom is carpeted

Child’s bedroom has an area rug

Bathroom was carpeted

Household smoking near window/garage, early life

Infant’s birthweight

Child’s month of birth

Paternal ethnicity

Frequency of taking folate supplements

Household smoking inside, early life

Mother takes folate supplements

Child’s sex

Kitchen is carpeted

**Figure 2a: Variable importance plot for Cotinine concentration**
Variables with higher importance scores (top right) were found through random forest regression to be more important in predicting the distribution of the log-transformed Cotinine concentration (ng/mL) than those with lower variable importance scores (leftwards). Variables known to relate to second-hand smoke were identified in green, while variables relating to household characteristics and reservoirs of thirdhand smoke are blue, and other variables are labelled red.


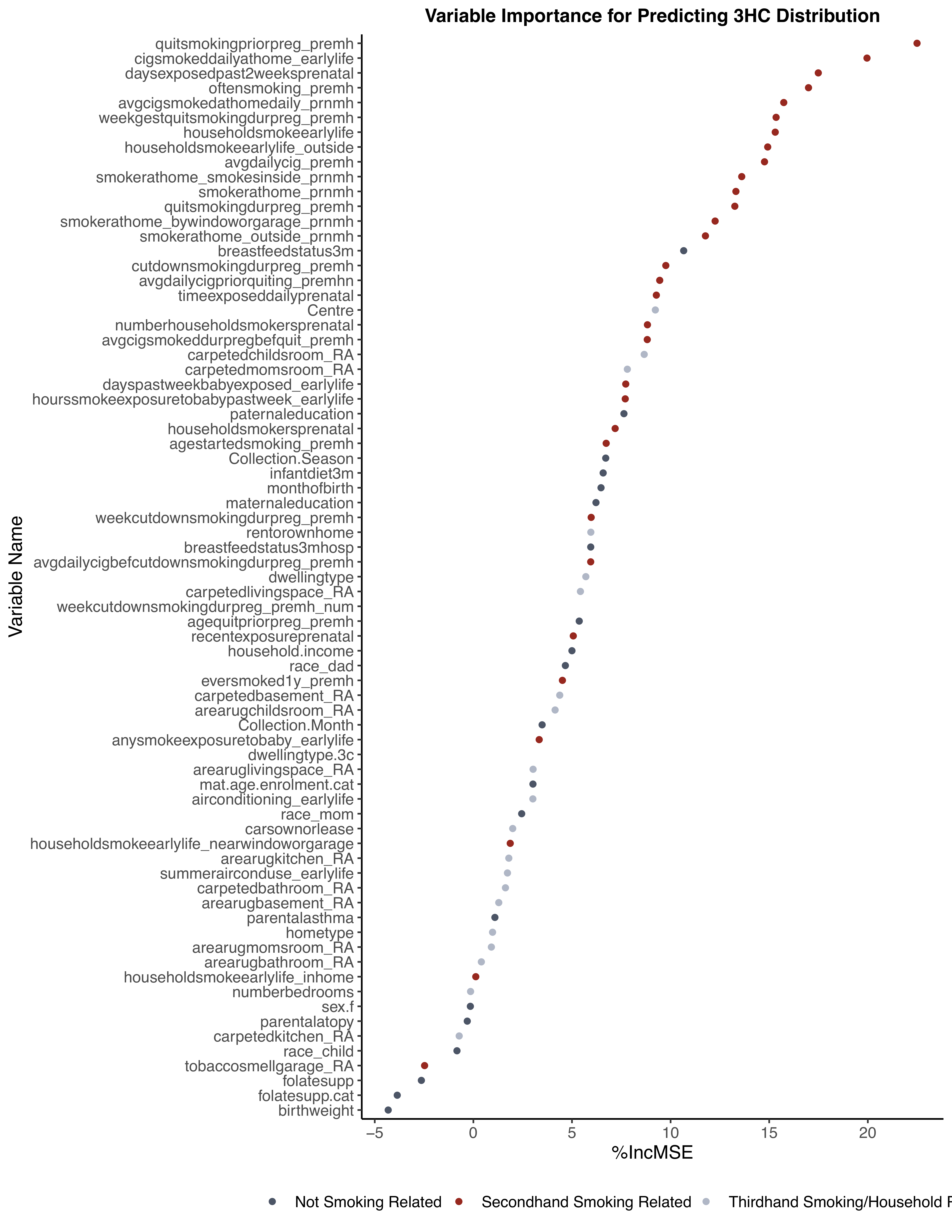


Maternal Smoking status prior to pregnancy
Cigarettes smoked daily at the home, early life

Days mother exposed to smoke during pregnancy, past 2 wks

How often did the mother smoke during pregnancy

Cigarettes smoked daily at the home, during pregnancy

Week Gestation that mother quit smoking

Household smoking in early life

Household smoking outside, early life

Cigarettes smoked daily by mother during pregnancy

Household smoking inside during pregnancy

Household smoking during pregnancy

Mother quit smoking during pregnancy

Household smoking near window/garage during pregnancy

Household smoking outside during pregnancy

Breastfeeding status at 3 months

Mother reduced smoking during pregnancy

Cigarettes smoked during pregnancy pre-quitting

Hours pregnant mother is exposed daily

Study Centre

Number of smokers at the home during pregnancy

Cigarettes smoked daily prior to quitting in pregnancy

Child’s bedroom is carpeted

Mom’s bedroom is carpeted

Days infant exposed to smoke in past week, 3mo

Hours infant exposed to smoke in past week, 3mo

Father’s highest level of education

Household smoker present during pregnancy

Age that the mother started smoking

Season at sample collection

Infant diet

Child’s month of birth

Mother’s highest level of education

Week Gestation that mother reduced smoking (grouped)

Home ownership

Breastfeeding status, including in hospital

Cigarettes smoked daily prior to reducing, in pregnancy

Dwelling type, 6 groups

Primary living space is carpeted

Week Gestation that mother reduced smoking(numeric)

Age mother quit smoking prior to pregnancy

Any recent smoke exposure during pregnancy

Household Income

Paternal ethnicity

Has the mother ever smoked for 1+ year?

Basement is carpeted

Child’s bedroom has an area rug

Month of sample collection

Any smoke exposure to baby since birth

Dwelling type, 3 groups

Living space has an area rug

Mother’s age at enrolment

Presence and type of air conditioning, early life

Maternal ethnicity

Number of cars owned or leased

Household smoking near window/garage, early life

Kitchen has an area rug

Air conditioner use during Summer months

Bathroom was carpeted

Basement has area rug

Parental history of asthmatic disease

Single or Multi-family home

Mom’s bedroom has an area rug

Bathroom has an area rug

Household smoking inside, early life

Number of bedrooms in the home

Child’s sex

Parental atopy status

Kitchen is carpeted

Child’s ethnicity (derived)

Tobacco smell detected in garage

Frequency of taking folate supplements

Mother takes folate supplements

Infant’s birthweight

**Figure 2b: Variable importance plot for *trans*-3’-Hydroxycotinine concentration**

Variables with higher importance scores (top right) in predicting the distribution of the log-transformed *trans*-3’-hydroxycotinine concentration (ng/mL) were found through random forest regression. Variables related to second-hand smoke (green), household characteristics or third-hand smoke reservoirs (blue), and other (red).

**Table 2: Variable Importance and R^2^ Scores for Potential Predictors**

|  | Outcome: Cotinine | | Outcome: *trans*-3’-Hydroxycotinine | |
| --- | --- | --- | --- | --- |
| **Predictor** | **Variable Importance Score (%MSE)** | **R^2^ from Bivariate Analysis (%)** | **Variable Importance Score (%MSE)** | **R^2^ from Bivariate Analysis (%)** |
| Maternal Smoking Status Prior to Pregnancy | 23.10 | 18.8 | 22.49 | 22.9 |
| Cigarettes smoked daily at home in early life | 18.92 | 14.4 | 19.96 | 18.3 |
| Frequency of maternal smoking during pregnancy | 18.70 | 16.0 | 16.99 | 18.1 |
| Days pregnant mother has been exposed to tobacco smoke in the past 2 weeks | 18.47 | 15.3 | 17.49 | 18.1 |
| Week of gestation mother quit smoking during pregnancy | 16.93 | 9.3 | 15.35 | 11.8 |
| Average Cigarettes Smoked in Pregnancy | 16.15 | 15.6 | 14.76 | 17.6 |
| Average Daily Cigarettes Smoked at Home in Pregnancy | 14.13 | 14.3 | 15.74 | 17.7 |
| Household Smoking since birth | 13.56 | 11.0 | 15.31 | 15.3 |
| Maternal Smoking Status during pregnancy | 11.44 | 14.8 | 13.26 | 18.6 |
| Average Daily Cigarettes during Pregnancy before quitting | 11.07 | 6.9 | 8.82 | 9.1 |
| Household Smoking during pregnancy | 11.06 | 11.3 | 13.31 | 14.6 |
| Household smoking indoors during pregnancy | 10.96 | 12.5 | 13.61 | 15.8 |
| Household smoking outside since birth | 10.69 | 8.0 | 14.92 | 11.5 |
| Household smoking outside during pregnancy | 10.54 | 11.8 | 11.77 | 15.1 |
| Household smoking near window or in garage during pregnancy | 10.53 | 11.5 | 12.26 | 14.9 |
| Household Income | 9.74 | 4.6 | 5.00 | 5.4 |
| Breastfeeding Status | 8.93 | 0.5 | 10.66 | 0.4 |
| Number of Household Smokers, Prenatal | 8.33 | 6.6 | 8.83 | 8.2 |
| Hours of exposure, past 2 weeks of pregnancy | 8.25 | 4.3 | 9.28 | 5.4 |
| Centre | 8.19 | 1.1 | 9.23 | 2.3 |
| Frequency of a household smoker in pregnancy | 7.79 | 6.5 | 7.19 | 8.2 |
| Cut down smoking during pregnancy | 7.52 | 15.0 | 9.76 | 18.1 |
| Dwelling Type, 7 groups | 7.46 | 1.6 | 5.70 | 1.8 |
| Days baby exposed in past week | 7.44 | 3.3 | 7.23 | 4.7 |
| Parental Asthma | 7.32 | 0.2 | 1.09 | 0.1 |
| Daily cigarettes before cutting down in pregnancy | 6.99 | 11.5 | 5.95 | 13.0 |
| Area rug in basement | 6.62 | 0.8 | 1.29 | 0.6 |
| Week Reduced Smoking during pregnancy | 5.95 | 11.9 | 5.97 | 13.4 |
| Paternal Education | 5.77 | 5.3 | 7.63 | 7.1 |
| Age Started Smoking | 5.53 | 7.0 | 6.73 | 9.6 |
| Recent prenatal smoke exposure | 5.51 | 8.4 | 5.07 | 10.6 |
| Dwelling Type, 3 groups | 5.38 | 1.2 | 3.08 | 1.3 |
| Number of Bedrooms | 5.35 | 0.2 | -0.14 | 0.4 |
| Carpeted basement | 5.30 | 0.8 | 4.38 | 0.6 |
| Carpeted living space | 5.28 | 0.1 | 5.43 | 0.03 |
| Hospital Breastfeeding Status | 5.15 | 0.5 | 5.95 | 0.5 |
| Infant Diet | 4.86 | 0.6 | 6.58 | 0.7 |
| Week gestation reduced smoking, numeric | 4.86 | 8.3 | 5.39 | 9.6 |
| Maternal Age | 4.83 | 3.5 | 3.02 | 4.7 |
| Hours baby exposed in past week of life | 4.65 | 3.2 | 7.70 | 4.6 |
| Air conditioning | 4.35 | 1.3 | 3.02 | 1.7 |
| Age quit prior to pregnancy | 4.12 | 0.2 | 5.37 | 0.3 |
| Home Ownership | 3.75 | 2.5 | 5.96 | 2.3 |
| Carpeted child’s room | 3.67 | 1.0 | 8.66 | 1.5 |
| Ever smoked for 1 year | 3.52 | 6.6 | 4.51 | 9.4 |
| Average cigarettes prior to quitting | 3.29 | 0.2 | 9.45 | 0.4 |
| Area rug in kitchen | 3.01 | <0.001 | 1.80 | 0.04 |
| Home Type | 2.95 | 0.2 | 0.97 | 0.2 |
| Collection Season | 2.93 | 0.2 | 6.71 | 0.3 |
| Maternal Education | 2.75 | 3.8 | 6.22 | 5.2 |
| Number of cars | 2.72 | 0.8 | 1.99 | 1.2 |
| Summer air conditioning | 2.51 | 0.04 | 1.73 | 0.1 |
| Any exposure to baby | 2.30 | 2.6 | 3.38 | 4.4 |
| Collection Month | 1.88 | 0.7 | 3.49 | 0.8 |
| Area rug in mom’s bedroom | 1.80 | <0.001 | 0.91 | 0.03 |
| Tobacco Smell in Garage | 1.78 | 1.9 | -2.47 | 2.5 |
| Maternal ethnicity | 1.67 | 2.3 | 2.45 | 3.4 |
| Area rug in living space | 1.42 | 0.09 | 3.03 | 0.3 |
| Parental Atopy | 1.40 | 0.05 | -0.31 | 0.2 |
| Area rug in bathroom | 1.32 | 0.1 | 0.41 | 0.4 |
| Child’s ethnicity | 1.26 | 1.4 | -0.83 | 2.5 |
| Carpeted mom’s room | 1.19 | 0.06 | 7.80 | 0.2 |
| Area rug in child’s room | 0.90 | 1.0 | 4.14 | 1.6 |
| Carpeted bathroom | 0.90 | <0.001 | 1.63 | <0.001 |
| Household smoking near a window or in the garage, early life | 0.75 | 3.5 | 1.87 | 4.7 |
| Birthweight | 0.66 | 0.07 | -4.32 | <0.001 |
| Month of birth | -0.06 | 0.4 | 6.47 | 0.6 |
| Paternal ethnicity | -0.29 | 1.6 | 4.66 | 3.1 |
| Folate Supplements, numeric | -0.71 | 0.02 | -2.64 | <0.001 |
| Household smoking inside, early life | -0.76 | 1.5 | 0.12 | 1.9 |
| Folate Supplements | -0.91 | 0.06 | -3.86 | 0.06 |
| Child’s sex | -1.16 | 0.03 | -0.15 | <0.001 |
| Carpeted kitchen | -2.92 | 0.1 | -0.72 | <0.001 |

Predictor Variables are ordered from highest (top) to lowest (bottom) variable importance against log-transformed cotinine concentration, as determined through random forest regression. Importance scores are reported to the nearest 2 decimal places and reflect either the percentage of increase in mean squared error (%MSE) the average model will incur should the predictor be excluded from the model. R^2^ values are reported as a percentage based on bivariate linear regression analysis between a predictor variable and the log-transformed metabolite concentration.

**
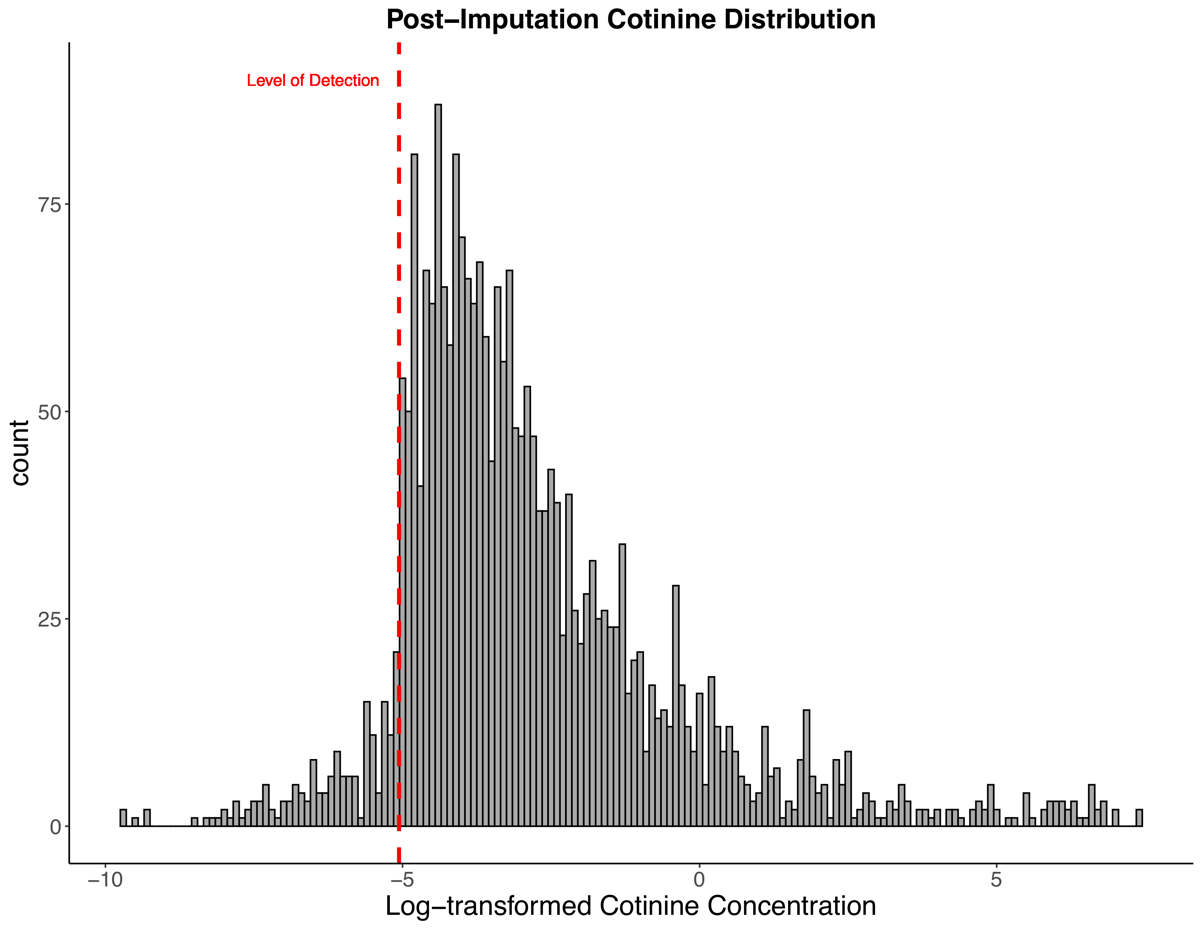
**

**Figure 3a: Distribution of log-transformed Cotinine concentration following imputation of concentrations below detection**

The post-imputation distribution ranges from -10.49 to 7.41, which equates to an original concentration range from 0.0007 to 170.0 ng/mL. The imputed cotinine concentrations ranged from -10.49 to -5.06 which equates to an original concentration of 0.0007 to 0.02997 ng/mL, consistent with our range of 0-0.03ng/mL. The Median concentration of the original cotinine concentrations was 0.016 ng/mL, with a mean of 0.0161 ng/mL. The level of detection was 0.03 ng/mL.

**A note on level of detection (LOD) versus level of quantification (LOQ)**

Some may want specification around the differentiation between the level of detection and level of quantification of a biomarker, while others use them interchangeably. Urine samples collected by the CHILD Cohort Study were analysed by a laboratory run by the Centre for Disease Control (CDC). This lab ran analysis to quantify the concentrations of our metabolites of interest. The CDC only provides concentration values at or above what they considered to be the lowest level of reliable detection. This lowest reportable concentration was deemed to be 0.03 ng/mL for Cotinine and 3HC. While their technology may be able to detect a concentration less than 0.03 ng/mL, these concentrations are too variable and do not meet their accuracy standards. For this reason, samples with concentration values below the level of reportable concentration were not provided to researchers and instead were flagged. For the analyses in this manuscript, concentrations below the level of detection (LOD) were imputed with a concentration between 0 and the LOD using the log-Normal distribution of the truncated concentration. For simplicity, ‘Level of Detection’ is the terminology that has been used in the manuscript.

**
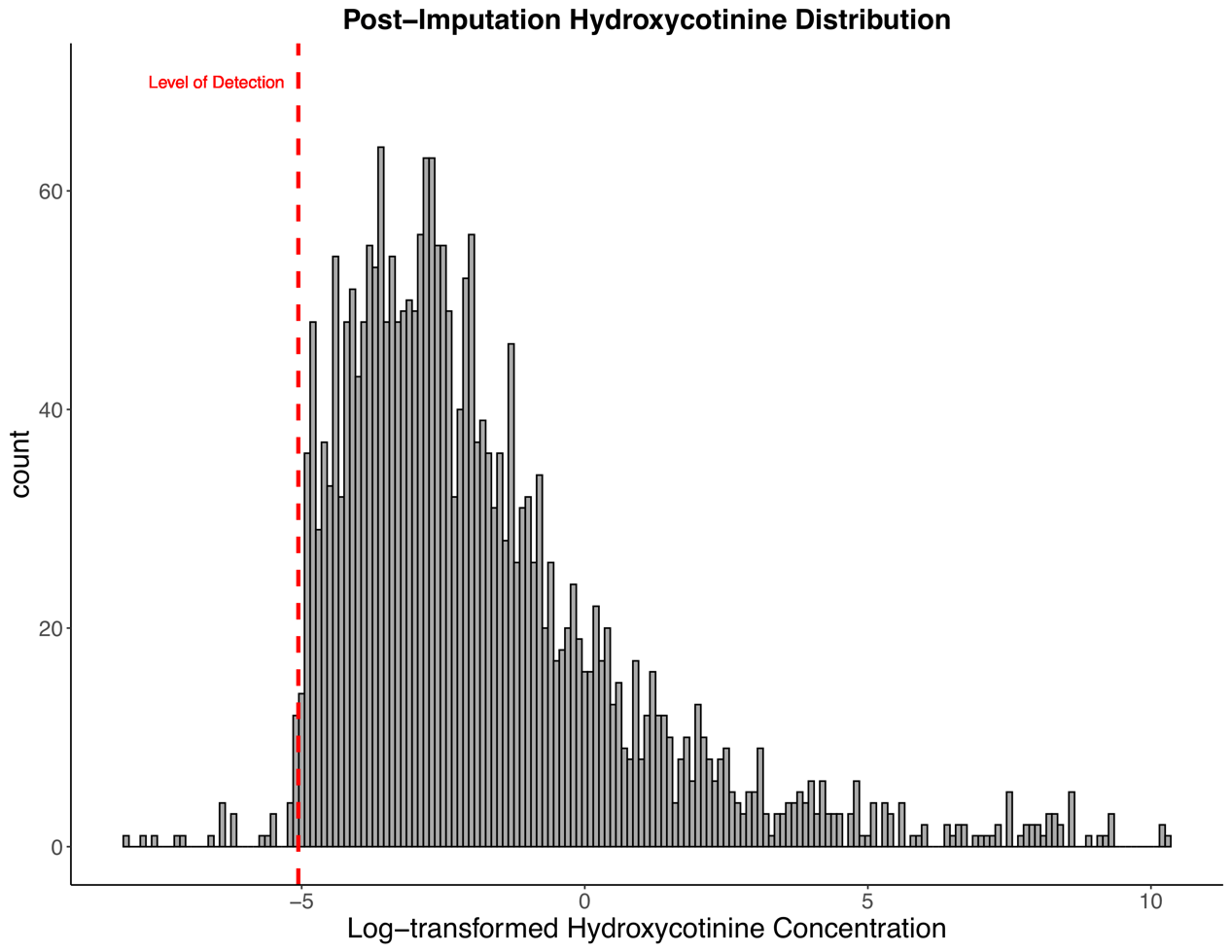
**

**Figure 3b: Distribution of log-transformed Hydroxycotinine concentration following imputation of concentrations below detection**

The post-imputation distribution ranges from -8.49 to 10.30, which equates to an original concentration range from 0.0028 to 1260.0 ng/mL. The imputed hydroxycotinine concentrations ranged from a raw concentration of 0.0004 to 0.0299 ng/mL, consistent with our range of 0-0.03ng/mL. The Median concentration of the original cotinine concentrations was 0.0141 ng/mL, with a mean of 0.0150 ng/mL. The level of detection was 0.03 ng/mL.

**Table 3: Summary statistics of each metabolite with and without dilution correction**

| **Metabolite** | **10^th^ %** | **25^th^ %** | **Median** | **Mean** | **75^th^ %** | **90^th^ %** | **SD** | **Geometric mean (95% CI)** |
| --- | --- | --- | --- | --- | --- | --- | --- | --- |
| **Cotinine*** | 0.02 | 0.04 | 0.08 | 1.87 | 0.23 | 0.77 | 13.73 | 0.12 (0.11-0.13) |
| **Cotinine^d^** | 0.03 | 0.05 | 0.09 | 1.81 | 0.25 | 0.97 | 11.00 | 0.13 (0.12-0.14 |
| **3HC*** | 0.04 | 0.07 | 0.16 | 6.67 | 0.45 | 1.90 | 67.84 | 0.22 (0.21-0.24) |
| **3HC^d^** | 0.05 | 0.08 | 0.17 | 7.47 | 0.51 | 2.30 | 61.57 | 0.25 (0.24-0.27) |

*Corrected for specific gravity and with concentrations imputed below the level of detection.

**^d^** Concentrations were not corrected for dilution but those below the level of detection were imputed.

Cotinine and 3HC are measured in units of ng/mL. SD = standard deviation; % = percentile of distribution range.

**Table 4: Predictor data dictionary**

| **Predictor** | **Description** | **Levels or range of responses** | **Collection method** |
| --- | --- | --- | --- |
| Maternal Smoking Status Prior to Pregnancy | The mother’s smoking status prior the pregnancy | 3 groups;   - Never smoked - Quit prior to the pregnancy - Did not quit prior to the pregnancy | Prenatal Maternal Health Questionnaire |
| Frequency of maternal smoking during pregnancy | How often the mother smoked during her pregnancy | 2 levels;   - Never - Occasionally or Regularly | Prenatal Maternal Health Questionnaire |
| Days pregnant mother has been exposed to tobacco smoke in the past 2 weeks | Average number of days the pregnant mother was exposed to tobacco smoke in the past 2 weeks | Numerical range from 0-14. | Home Environment Questionnaire, completed during pregnancy |
| Week of gestation mother quit smoking during pregnancy | Week of gestation that the mother quit smoking during her pregnancy | Numerical range from 0-25. | Prenatal Maternal Health Questionnaire |
| Average Cigarettes Smoked in Pregnancy | Average number of cigarettes the pregnancy mother smoked per day | 3 groups;   - None - Less than 10/day - 10 to 20/day | Prenatal Maternal Health Questionnaire |
| Average Daily Cigarettes Smoked at Home in Pregnancy | The average number of cigarettes/cigars/pipes smoked at the household per day | 5 groups;   - None - Less than 1/day - 1-5/day - 6-10 /day - More than 10/day | Prenatal Maternal Health Questionnaire |
| Household Smoking since birth | Whether or not anyone smokes at the home since the child’s birth | 2 levels;   - No - Yes | Home Environment Questionnaire, completed by parent(s) at 3-4 months of age |
| Average Daily Cigarettes during Pregnancy before quitting | Average number of cigarettes the pregnancy mother smoked per day prior to quitting | 4 groups;   - Never Smoke - Less than 5/day - 5-10/day - 10-25 /day | Prenatal Maternal Health Questionnaire |
| Household Income | Parental response to “What is the best estimate of total income, before taxes and deductions, of all household members, from all sources in the past 12 months.” | 5 levels;   - $0-49,999/year - $50,000-99,999/year - $100,000-149,999/year - $150,000+/year - Prefers not to say | Socioeconomic Status Questionnaire*  *completed during pregnancy |
| Breastfeeding Status | Status of infants breastfeeding at 3 months of age | 3 levels;   - Not breastfed (no recorded initiation of breastfeeding in hospital) - Partially breastfed (any combination of breastfeeding and supplementation) - Exclusively breastfed until any supplement (including formula, other fluid (non-human milk, juice) or food) is introduced.) | Determined using a combination of the birth and nutrition questionnaires |
| Centre | Study Centre location of the subject | 4 groups;   - Vancouver - Edmonton - Winnipeg, Morden, and Winkler - Toronto | Home Environment Questionnaire completed by parents when child is 3-4 months of age |
| Frequency of a household smoker in pregnancy | Whether and how frequent anyone smoked inside the home during pregnancy | 3 levels;   - None - Occasional - Regular | Home Environment Questionnaire, completed during pregnancy |
| Parental Asthma | Whether or not at least one parent has a history or current diagnosis of asthma. If one parent was missing, and the other had a positive response, the child was positive. If both had no response, or if one was a no and the other missing, the child did not have a history. | 2 levels;   - No - Yes   If both parents' results were missing, the child was not assigned a response. | Derived from Prenatal Health Questionnaire |
| Paternal Education | Highest level of education achieved by the father | 4 levels;   - Highschool or less - Some post-secondary - Completed post-secondary - Masters of PhD | Socioeconomic Status Questionnaire*  *completed during pregnancy |
| Dwelling Type, 3 groups | The type of dwelling that best described the subject’s home | 3 groups;   - Mobile home/trailer/Other - Single Family (detached or semi-detached) - Multi-family/Apartment (any number of levels) | Socioeconomic Status Questionnaire*  *completed during pregnancy |
| Week gestation reduced smoking | Week of gestation that the mother reduced smoking during her pregnancy | Numeric range from 0-21 | Prenatal Maternal Health Questionnaire |
| Home Ownership | Questionnaire response to “Does your family own or rent your house/apartment?” | 2 levels;   - Rent - Own | Socioeconomic Status Questionnaire*  *completed during pregnancy |
| Carpeted child’s room | Research staff indication of whether or not the child’s bedroom had installed carpet as the type of flooring. | 2 levels;   - No - Yes - No child-specific room | Home assessment Questionnaire, completed by research staff at 3-4 months of age |
| Collection Season | Season of urine sample collection. Derived from the month when urine samples were collected. | 4 levels, set as per Northern Meteorological Seasons;   - Spring - Summer - Fall - Winter | Urinary biomarker sampling collected at the 3-4month of age home assessment |

This table offers an explanation of how each of the variables in our final predictor models (see Tables A5a-b) were organized and derived.

**Table 5a. Cotinine Multivariable Linear Regression Model and Multiplicative Change in log-transformed Cotinine concentrations**

| **Predictor variables (n=13)** | **Cotinine Model Coefficients  (95% CI)** | **Multiplicative Change in Concentration**  **(95% CI)** | **R^2^ (%) of Model** | **Multivariable Cotinine Model Coefficients  (95% CI)*** | **Multiplicative Change in Concentration (95% CI)*** | **Model R^2*^ (%) 31.43** |
| --- | --- | --- | --- | --- | --- | --- |
| **Mother’s Smoking Prior to Pregnancy** |  |  | 18.83 |  |  |  |
| Did not quit smoking prior to pregnancy vs. Never Smoked | 4.30 (3.91, 4.69) | 19.72 (15.05-24.86) |  | 1.24 (0.58, 1.90) | 2.2.36 (1.49-3.74) |  |
| Quit Smoking prior to pregnancy vs. Never Smoked | 0.42 (0.18, 0.67) | 1.34 (1.13-1.59) |  | 0.17 (-0.06, 0.40) | 1.13 (0.96-1.32) |  |
| **Mother’s Reported Smoking Frequency in Pregnancy** | |  | 16.0 |  |  |  |
| Smoked daily or occasionally vs. Never Smoked | 4.30 (3.87, 4.74) | 19.76 (14.66-26.64) |  | 2.20 (1.53, 2.87) | 4.59 (2.89-7.29) |  |
| **Days Exposed to Cigarette Smoke in past 2 weeks during pregnancy** | |  | 15.25 |  |  |  |
| Continuous | 0.29 (0.26, 0.32) | 1.22 (1.20-1.25) |  | 0.07 (0.03, 0.10) | 1.05 (1.02-1.07) |  |
| **Week of gestation when mother quit smoking** | |  | 7.79 |  |  |  |
| Continuous | 0.33 (0.28, 0.38) | 1.26 (1.21-1.30) |  | 0.14 (0.08, 0.21) | 1.10 (1.05-1.15) |  |
| **Week of gestation when mother cut down smoking** | |  | 8.31 |  |  |  |
| Continuous | 0.49 (0.42, 0.56) | 1.40 (1.34-1.47) |  | -0.08 (-0.17, -0.01) | 0.95 (0.89-1.01) |  |
| **Average Daily Cigarettes Smoked at the Home during pregnancy** | |  | 14.27 |  |  |  |
| Less than 1/day vs None | 3.63 (3.11, 4.15) | 12.41 (8.66-17.79) |  | 0.64 (0.03, 1.25) | 1.56 (1.02-2.37) |  |
| 1-5/day vs None | 0.33 (-0.18, 0.83) | 1.25 (0.89-1.78) |  | -0.54 (-1.03, -0.06) | 0.69 (0.49-0.96) |  |
| 6-10/day vs None | 0.38 (-0.23, 0.99) | 1.30 (0.85-1.98) |  | 0.15 (-0.40, 0.70) | 1.11 (0.76-1.63) |  |
| 11+/day vs None | -0.19 (-0.74, 0.35) | 0.88 (0.60-1.28) |  | -0.11 (-0.61, 0.39) | 0.93 (0.66-1.31) |  |
| **Has anyone smoked at the baby’s home since their birth?** | |  | 10.97 |  |  |  |
| Yes vs. No | 2.39 (2.10, 2.69) | 5.26 (4.28-6.46) |  | 0.31 (-0.19, 0.80) | 1.24 (0.88-1.74) |  |
| **Average Daily Cigarettes Smoked at the Home since child’s birth** | |  | 14.37 |  |  |  |
| 1-5/day vs. None | 3.61 (3.07, 4.15) | 12.23 (8.41, 17.77) |  | 0.77 (0.10, 1.43) | 1.70 (1.08, 2.69) |  |
| 6-10/day vs. None | -0.48 (-1.05, 0.08) | 0.72 (0.48, 1.06) |  | 0.03 (-0.54, 0.60) | 1.02 (0.69, 1.51) |  |
| 11+/day vs. None | 0.01 (-0.57, 0.60) | 1.01 (0.67, 1.51) |  | 0.17 (-0.38, 0.71) | 1.12 (0.77, 1.64) |  |
| **Breastfeeding status at 3 months** |  |  | 0.48 |  |  |  |
| Partially vs. Exclusively Breastfed | 0.36 (0.13, 0.59) | 1.29 (1.10-1.51) |  | -0.28 (-0.48, -0.08) | 0.82 (0.72-0.95) |  |
| Never vs. Exclusively Breastfed | 0.13 (-0.08, 0.34) | 1.10 (0.95-1.27) |  | -0.09 (-0.27, 0.09) | 0.94 (0.83-1.07) |  |
| **Dwelling Type** |  |  | 1.23 |  |  |  |
| Multi-family vs. single-family Home | 0.83 (0.18, 1.47) | 1.77 (1.13-2.77) |  | 0.19 (-0.36, 0.75) | 1.14 (0.78-1.68) |  |
| Trailer/other vs. single-family Home | 0.04 (-0.37, 0.44) | 1.03 (0.77-1.36) |  | -0.16 (-0.51, 0.19) | 0.89 (0.70-1.14) |  |
| **Household Income** |  |  | 4.60 |  |  |  |
| $50,000-99,999/year vs. <$50,000/year | -0.96 (-1.26, -0.65) | 0.52 (0.42-0.64) |  | -0.45 (-0.72, -0.18) | 0.73 (0.61-0.88) |  |
| $100,000-149,999/year vs. <$50,000/year | 0.90 (0.62, 1.18) | 1.87 (1.54-2.27) |  | 0.31 (-0.06, 0.56) | 1.24 (1.04-1.47) |  |
| $150,000+/year vs. <$50,000/year | 0.18 (-0.05, 0.41) | 1.13 (0.97-1.33) |  | 0.08 (-0.12, 0.27) | 1.05 (0.92-1.21) |  |
| Prefers not to say vs. <$50,000/year | 0.06 (-0.14, 0.26) | 1.04 (0.91-1.20) |  | -0.06 (-0.23, 0.11) | 0.96 (0.85-1.08) |  |
| **Paternal Education** |  |  | 5.29 |  |  |  |
| Some post-secondary vs. Highschool or less | -1.37 (-1.63, -1.11) | 0.39 (0.32-0.46) |  | -0.37 (-0.62, -0.13) | 0.77 (0.65-0.92) |  |
| Completed post-secondary vs. Highschool or less | 0.13 (-0.11, 0.36) | 1.09 (0.93-1.29) |  | -0.16 (-0.37, 0.04) | 0.89 (0.77-1.03) |  |
| Masters or PhD vs. Highschool or less | 0.04 (-0.16, 0.25) | 1.03 (0.89-1.19) |  | 0.05 (-0.13, 0.23) | 1.04 (0.92-1.17) |  |
| **Parental History of Asthma** |  |  | 0.25 |  |  |  |
| Yes vs. No | 0.25 (0.03, 0.47) | 1.19 (1.02-1.39) |  | 0.22 (0.03, 0.41) | 1.16 (1.02-1.32) |  |

Estimated change in log-transformed metabolite level by predictors in unadjusted regression models (95% confidence intervals), as well as the coinciding R^2^ values are shown to the nearest second decimal place.

* Estimated change in log-transformed metabolite level by predictors in **adjusted** final regression models (95% confidence intervals), as well as the coinciding R^2^ values are shown to the nearest second decimal place**.**

**Table 5b. *Trans*-3’-Hydroxycotinine Multivariable Linear Regression Model and Multiplicative Change in log-transformed *trans*-3’-Hydroxycotinine concentrations**

| **Predictor variables (n=19)** | **3HC Model  Coefficients  (95% CI)** | **Multiplicative Change in Concentration**  **(95% CI)** | **R^2^ (%) of model** | **Multivariable 3HC Model  Coefficients  (95% CI)*** | **Multiplicative Change in Concentration (95% CI)** | **Model R^2*^ (%)**  **40.89** |
| --- | --- | --- | --- | --- | --- | --- |
| **Mother’s Smoking Prior to Pregnancy** |  |  | 22.90 |  |  |  |
| Did not quit smoking prior vs. Never Smoked | 4.95 (4.55, 5.35) | 30.86 (23.40-40.68) |  | 1.99 (1.09, 2.88) | 3.97 (2.13-7.37) |  |
| Quit Smoking prior vs. Never Smoked | 0.68 (0.43, 0.93) | 1.60 (1.35-1.90) |  | 0.45 (0.23, 0.68) | 1.37 (1.17-1.60) |  |
| **Average Cigarettes Smoked Daily Prior to Quitting During Pregnancy** | |  | 9.11 |  |  |  |
| Less than 5/day vs. Never Smoked | 3.61 (2.91, 4.31) | 12.20 (7.49-19.88) |  | 0.65 (-0.32, 1.62) | 1.57 (0.80-3.07) |  |
| 5 to 10/day vs. vs. Never Smoked | -0.54 (-1.31, 0.23) | 0.69 (0.40-1.17) |  | 1.60 (0.78, 2.43) | 3.03 (1.72-5.38) |  |
| 11 to 25/day vs. Never Smoked | 0.50 (-0.32, 1.32) | 1.42 (0.80-2.50) |  | -0.21 (-0.91, 0.49) | 0.86 (0.53-1.40) |  |
| **Mother’s Reported Smoking Frequency in Pregnancy** | |  | 18.11 |  |  |  |
| Smoked daily or occasionally vs. Never Smoked | 4.80 (4.35, 5.24) | 27.77 (20.39-37.81) |  | 3.13 (1.16, 5.10) | 8.73 (2.22-34.21) |  |
| **Average Cigarettes Smoked by Mother in early pregnancy** | |  | 17.56 |  |  |  |
| Less than 10/day vs. None | 5.39 (4.67, 6.11) | 42.01 (25.51-69.20) |  | -0.60 (-2.72, 1.53) | 0.66 (0.15-2.88) |  |
| 10-20/day vs. None | -1.97 (-2.75, -1.18) | 0.26 (0.15-0.44) |  | 1.58 (0.25, 2.91) | 2.98 (1.19-7.50) |  |
| **Days Exposed to Cigarette Smoke in past 2 weeks during pregnancy** | |  | 18.08 |  |  |  |
| Continuous | 0.33 (0.30, 0.36) | 1.26 (1.23-1.29) |  | 0.05 (0.01, 0.10) | 1.04 (1.01-1.07) |  |
| **Mother had any recent tobacco smoke exposure during pregnancy** | |  | 10.58 |  |  |  |
| Recent exposure | 1.98 (1.73, 2.23) | 3.94 (3.31-4.68) |  | 0.24 (-0.07, 0.54) | 1.17 (0.95-1.46) |  |
| **Week of gestation when mother quit smoking** |  |  | 9.80 |  |  |  |
| Continuous | 0.39 (0.34, 0.44) | 1.30 (1.26-1.35) |  | 0.16 (0.08, 0.25) | 1.12 (1.06-1.19) |  |
| **Did anyone smoke at the baby’s home during pregnancy?** | | 8.91 |  |  |  |  |
| Occasionally vs. None | 3.54 (2.74, 4.33) | 11.60 (6.68-20.12) |  | 0.16 (-0.54, 0.87) | 1.12 (0.69-1.83) |  |
| Regularly vs. None | 4.93 (4.00, 5.86) | 30.41 (15.87-58.25) |  | 0.54 (-0.37, 1.44) | 1.45 (0.78-2.71) |  |
| **Average Daily Cigarettes Smoked at the Home during pregnancy** | |  | 17.69 |  |  |  |
| Less than 1/day vs None | 4.07 (3.54, 4.61) | 16.85 (11.64-24.37) |  | 0.21 (-0.44, 0.86) | 1.16 (0.74-1.81) |  |
| 1-5/day vs None | 0.24 (-0.28, 0.75) | 1.18 (0.82-1.69) |  | -0.85 (-1.36, -0.34) | 0.56 (0.39-0.79) |  |
| 6-10/day vs None | 0.61 (-0.02, 1.23) | 1.52 (1.00-2.34) |  | 0.23 (-0.33, 0.79) | 1.17 (0.79-1.73) |  |
| 11+/day vs None | -0.13 (-0.69, 0.43) | 0.91 (0.62-1.34) |  | 0.09 (-0.40, 0.58) | 1.06 (0.76-1.50) |  |
| **Has anyone smoked at the baby’s home since their birth?** | |  | 15.27 |  |  |  |
| Yes vs. No | 2.96 (2.65, 3.26) | 7.77 (6.30-9.60) |  | 0.70 (0.22, 1.19) | 1.63 (1.16-2.28) |  |
| **Average Daily Cigarettes Smoked at the Home since child’s birth** | |  | 18.32 |  |  |  |
| 1-5/day vs. None | 4.03 (3.48, 4.58) | 16.37 (11.17-24.00) |  | 0.41 (-0.24, 1.07) | 1.33 (0.85-2.10) |  |
| 6-10/day vs. None | -0.80 (-1.37, -0.22) | 0.58 (0.39-0.86) |  | -0.12 (-0.68, 0.45) | 0.92 (0.62-1.36) |  |
| 11+/day vs. None | 0.11 (-0.49, 0.71) | 1.08 (0.71-1.63) |  | 0.24 (-0.30, 0.78) | 1.18 (0.81-0.72) |  |
| **Carpeting in the home** |  |  | 1.53 |  |  |  |
| Child’s room is not carpeted vs. No child-specific room | -0.59 (-0.81, -0.38) | 0.66 (0.57-0.77) |  | -0.18 (-0.37, -0.0) | 0.88 (0.78-1.00) |  |
| Child’s room is carpeted vs. No child-specific room | 0.27 (0.09, 0.46) | 1.21 (1.06-1.37) |  | -0.05 (-0.20, 0.11) | 0.97 (0.87-1.08) |  |
| **Breastfeeding status at 3 months** |  |  | 0.39 |  |  |  |
| Partially vs. Exclusively Breastfed | 0.32 (0.08, 0.56) | 1.25 (1.06-1.48) |  | -0.52 (-0.72, -0.32) | 0.70 (0.61-0.80) |  |
| Not vs. Exclusively Breastfed | 0.03 (-0.19, 0.25) | 1.02 (0.88-1.19) |  | -0.29 (-0.47, -0.12) | 0.82 (0.72-0.92) |  |
| **Dwelling Type** |  |  | 1.29 |  |  |  |
| Multi-family vs. single-family Home | 0.99 (0.31, 1.66) | 1.98 (1.24-3.17) |  | 0.30 (-0.25, 0.84) | 1.23 (0.84-1.79) |  |
| Trailer/other vs. single-family Home | 0.12 (-0.31, 0.54) | 1.08 (0.81-1.46) |  | -0.23 (-0.59, 0.12) | 0.85 (0.66-1.09) |  |
| **Home Ownership** |  |  | 2.31 |  |  |  |
| Rents vs. Owns Home | 0.89 (0.64, 1.15) | 1.86 (1.56-2.21) |  | -0.02 (-0.26, 0.22) | 0.99 (0.84-1.17) |  |
| **Household Income** |  |  | 5.42 |  |  |  |
| $50,000-99,999/year vs. <$50,000/year | -0.93 (-1.25, -0.61) | 0.52 (0.42-0.65) |  | -0.34 (-0.62, -0.07) | 0.79 (0.65-0.95) |  |
| $100,000-149,999/year vs. <$50,000/year | 1.18 (0.89, 1.47) | 2.26 (1.85-2.77) |  | 0.38 (0.13, 0.64) | 1.31 (1.09-1.56) |  |
| $150,000+/year vs. <$50,000/year | 0.22 (-0.02, 0.46) | 1.17 (0.99-1.37) |  | 0.05 (-0.14, 0.25) | 1.04 (0.91-1.19) |  |
| Prefers not to say vs. <$50,000/year | 0.24 (0.03, 0.45) | 1.18 (1.02-1.36) |  | 0.11 (-0.06, 0.28) | 1.08 (0.96-1.21) |  |
| **Collection Season** |  |  | 0.32 |  |  |  |
| Spring vs. Fall | -0.18 (-0.49, 0.12) | 0.88 (0.71-1.08) |  | -0.34 (-0.58, -0.10) | 0.80 (0.67-0.93) |  |
| Summer vs. Fall | 0.13 (-0.18, 0.45) | 1.10 (0.88-1.36) |  | 0.18 (-0.06, 0.43) | 1.13 (0.96-1.34) |  |
| Winter vs. Fall | -0.21 (-0.52, 0.11) | 0.87 (0.70-1.08) |  | -0.32 (-0.57, -0.07) | 0.80 (0.67-0.95) |  |
| **Study Centre** |  |  | 2.30 |  |  |  |
| Toronto vs. Edmonton | -0.50 (-0.83, -0.17) | 0.71 (0.56-0.89) |  | -0.11 (-0.40, 0.17) | 0.92 (0.76-1.12) |  |
| Vancouver vs. Edmonton | -0.82 (-1.14, -0.51) | 0.57 (0.45-0.70) |  | -0.59 (-0.86, -0.31) | 0.67 (0.55-0.80) |  |
| Winnipeg vs. Edmonton | 0.06 (-0.24, 0.37) | 1.05 (0.84-1.29) |  | -0.15 (-0.40, 0.10) | 0.90 (0.76-1.07) |  |
| **Paternal Education** |  |  | 7.05 |  |  |  |
| Some post-secondary vs. Highschool or less | -1.58 (-1.85, -1.31) | 0.33 (0.28-0.40) |  | -0.45 (-0.70, -0.20) | 0.73 (0.62-0.87) |  |
| Completed post-secondary vs. Highschool or less | 0.23 (0.02, 0.47) | 1.17 (0.99-1.39) |  | -0.11 (-0.31, 0.10) | 0.93 (0.81-1.07) |  |
| Masters or PhD vs. Highschool or less | 0.18 (-0.03, 0.40) | 1.14 (0.98-1.32) |  | 0.20 (0.25, 0.37) | 1.15 (1.02-1.30) |  |

Estimated change in log-transformed metabolite level by predictors in unadjusted regression models (95% confidence intervals), as well as the coinciding R^2^ values are shown to the nearest second decimal place.
* Estimated change in log-transformed metabolite level by predictors in **adjusted** final regression models (95% confidence intervals), as well as the coinciding R^2^ values are shown to the nearest second decimal place.

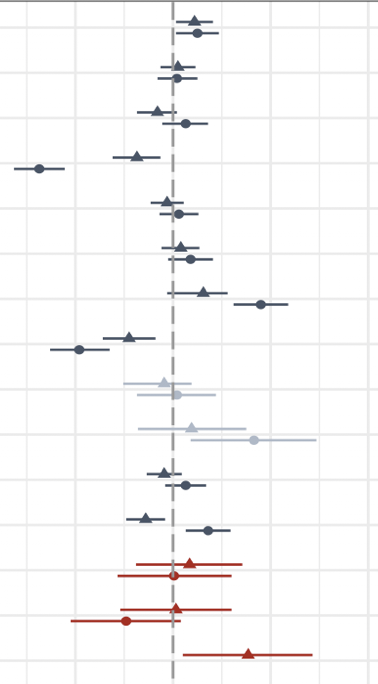

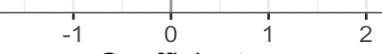

**Figure 4a. Multiplicative Change in Cotinine Multivariable Linear Regression Model**

Multiplicative change in urinary cotinine concentration (point) and 95% confidence intervals (line) are displayed for each variable in the prediction model calculated using the inverse-log-transformed coefficients. Variables related to second-hand smoke are shown in red, not smoking-related variables in blue, and variables related to household characteristics in grey. Intervals with a change estimate displayed as a circle are based on bivariate analysis between each predictor and urinary cotinine, while estimates displayed with a triangle reflect estimates from multivariable model.

**Figure 4b. Multiplicative Change in *trans*-3’-Hydroxycotinine Multivariable Linear Regression Model**

Multiplicative change in urinary *trans*-3’-hydroxycotinine concentration (point) and 95% confidence intervals (line) are displayed for each variable in the prediction model calculated using the inverse-log-transformed coefficients. Variables related to second-hand smoke are shown in red, not smoking-related variables in blue, and variables related to household characteristics in grey. Intervals with a change estimate displayed as a circle are based on bivariate analysis between each predictor and urinary *trans*-3’-hydroxycotinine, while estimates displayed with a triangle reflect estimates from multivariable model.
